# Photobiomodulation improves depression symptoms: a systematic review and meta-analysis of randomized controlled trials

**DOI:** 10.3389/fpsyt.2023.1267415

**Published:** 2024-01-31

**Authors:** Qipei Ji, Shichang Yan, Jilin Ding, Xin Zeng, Zhixiang Liu, Tianqi Zhou, Zhuorao Wu, Wei Wei, Huaqiang Li, Shuangyue Liu, Shuangchun Ai

**Affiliations:** ^1^School of Health Preservation and Rehabilitation, Chengdu University of Traditional Chinese Medicine, Chengdu, China; ^2^Department of Rehabilitation, Mianyang Hospital of Traditional Chinese Medicine, Mianyang, China; ^3^Department of Endocrinology and Metabolism, The Affiliated Hospital of Southwest Medical University, Luzhou, China

**Keywords:** photobiomodulation, t-PBM, s-PBM, low-level laser therapy, depression, sleep, meta-analysis

## Abstract

**Background:**

Depression is a common mental illness that is widely recognized by its lack of pleasure, fatigue, low mood, and, in severe cases, even suicidal tendencies. Photobiomodulation (PBM) is a non-invasive neuromodulation technique that could treat patients with mood disorders such as depression.

**Methods:**

A systematic search of ten databases, including randomized controlled trials (RCTs) for depression, was conducted from the time of library construction to September 25, 2023. The primary outcome was depression. The secondary outcome was sleep. Meta-analysis was performed using RevMan (version 5.4) and Stata (version 14.0). Subgroup analyses were performed to identify sources of heterogeneity. The certainty of the evidence was assessed using the Grading of Recommendations Assessment, Development, and Evaluation (GRADE).

**Results:**

Three thousand two hundred and sixty-five studies were retrieved from the database and screened for inclusion in eleven trials. The forest plot results demonstrated that PBM alleviated depression (SMD = −0.55, 95% CI [−0.75, −0.35], *I^2^* = 46%). But it is not statistically significant for patients’ sleep outcomes (SMD = −0.82, 95% CI [−2.41, 0.77], *I^2^* = 0%, *p* > 0.05). Subgroup analysis showed that s-PBM was superior to t-PBM in relieving symptoms of depression. The best improvement for t-PBM was achieved using a wavelength of 823 nm, fluence of 10–100 J/cm^2^, irradiance ≤ 50 mW/cm^2^, irradiance time of 30 min, treatment frequency < 3/week, and number of treatments > 15 times. The best improvement for s-PBM was achieved using a wavelength of 808 nm, fluence ≤1 J/cm^2^, irradiance of 50–100 mW/cm^2^, irradiance time ≤ 5 min, treatment frequency ≥ 3/week, number of treatments >15 times. All results had evidence quality that was either moderate or very low, and there was no bias in publication.

**Conclusion:**

We conclude that PBM is effective in reducing depression symptoms in patients. However, the current number of studies is small, and further studies are needed to extend the current analysis results.

**Systematic Review Registration:**

https://www.crd.york.ac.uk/PROSPERO/, CRD42023444677.

## 1. Introduction

Depression is a common mental illness that is widely recognized by its lack of pleasure, fatigue, low mood, and, in severe cases, even suicidal tendencies (1). As reported in 2017 statistics released by the World Health Organization (WHO), 322 million people worldwide suffer from depression, with an overall prevalence rate of 4.4%, including 5.1% for women and 3.6% for men (2). Depression can be a significant burden on an individual’s life, and those who suffer from it are more susceptible to developing cardiovascular disease, long-term diseases such as obesity, and other illnesses caused by lifestyle behaviors (e.g., smoking alcohol abuse) (3, 4). Furthermore, depression is a burden on society due to the 20 times higher than usual suicide rate (5, 6). Sleeping disorders, which are often caused by depression, are often positively correlated with mortality and have a negative impact on patients’ quality of life as the disease progresses (7–9). Pharmacological treatments such as tricyclic antidepressant treatment are still the mainstay of treatment for depression today. However, with less than half of all patients worldwide receiving antidepressant treatment [possibly due to side effects and low patient compliance (10, 11)], treating depression remains difficult and challenging (12).

Photobiomodulation (PBM), also known as low-level laser therapy (LLLT), represents a device-based intervention employing visible and/or near-infrared (NIR) light emitted by lasers or light-emitting diodes (LEDs) to regulate physiological processes (13–16). Our review examines the impact of PBM on brain physiology, with a specific focus on its effects on depression and sleep. The research on PBM’s influence on brain physiology can be broadly categorized by where the light is applied within the body: transcranial PBM (t-PBM) and systemic PBM (s-PBM) (15). t-PBM directly modulates brain physiology by delivering light to the brain (17). At the same time, s-PBM indirectly influences brain physiology by applying light to other body areas, such as acupoints or pain points, thereby inducing systemic physiological changes that subsequently affect brain physiology (13–16). The mechanism of action (MoA) for t-PBM has partially been elucidated - transcranial-delivered light is absorbed by mitochondria in brain cells, normalizing mitochondrial function and restoring energy homeostasis (18–20). Mitochondrial dysfunction has been liked to conditions such as depression (21), sleep disturbances (22), and cognitive impairment (23, 24). Under appropriate light irradiance and luminous flux, red and NIR photons (ranging from 600 nm to 1,164 nm), absorbed by cytochrome c oxidase (CCO) in brain cells, contribute to the normalization of mitochondrial function (25) and an increase in ATP synthesis (18, 26, 27). This, in turn, has beneficial effects on enhancing cognitive and emotional brain functioning (28–31). The precise mechanism (s) by which s-PBM could affect brain physiology is still unclear. However, the beneficial effects of s-PBM on neurons have been well established, even if it is applied to distant locations and not directly to neurons (32). s-PBM can activate circulating immune cells (13, 33–35) or stem cells (36), the mitochondria (37), and the cardiovascular or the lymphatic system, which then leads to an overall increase in the mitochondrial activity to increase (similar to direct stimulation with t-PBM), resulting in a neuroprotective effect (32). In summary, both t-PBM and s-PBM possess neuroprotective effects, and when comparing the two, direct stimulation with t-PBM is more effective, at least in animal models of Parkinson’s (38).

PBM is currently garnering considerable attention in neuropsychiatric disorders (39). Evidence indicates that t-PBM can ameliorate depressive symptoms (40), with potential mediation through enhanced cerebral blood flow (31). For instance, Schiffer et al. (31) found that people with major depressive disorder (MDD) complicated by anxiety experienced significant reductions in depression scores after 2 weeks of t-PBM treatment. Clinical studies have demonstrated that s-PBM irradiation of the back and thighs alleviates depressive symptoms in patients with low back pain (41). s-PBM irradiation of the neck and acupuncture points in patients with alcohol addiction also relieves depressive symptoms (42). The use of s-PBM has been shown to relieve depression in patients with low back pain.

The FDA has cleared PBM for peripheral pain, and it is gaining popularity as a neuromodulation technique (13). Specifically, PBM was utilized to target emotional disturbance in animal models and humans, and it was demonstrated to modify the neural inflammatory profile of rat brains (43–45).

Given the current global disease burden of depression and sleep disorders, few systematic review and Meta-analyses have explored the effects of PBM on depression (46, 47), and even fewer have explored the optimal dose of PBM. Therefore, we attempted to summarize the most compelling evidence from randomized controlled trials using meta-analysis to explore the feasibility of PBM for the treatment of depressive symptoms and to summarize the optimal dose of PBM for the treatment of depressive symptoms.

## 2. Methods

This study has been registered with PROSPERO, registration number CRD42023444677. It was carried out in accordance with the Preferred Reporting Items for Systematic Reviews and Meta-analyses (PRISMA) (Supplementary Appendix 1) (48).

### 2.1. Search strategy

From their inceptions to September 25, 2023, two reviewers independently searched PubMed, Embase, the Cochrane Library, Web of Science, ScienceDirect, Psyclnfo, CNKI, VIP, Sinomed, and Wanfang Database. Search terms included Low-Level Light Therapy, Photobiomodulation Therapy, transcranial photobiomodulation, near-infrared light, depression, and randomized controlled trials. In Supplementary Appendix 2, the search formula is displayed in detail. In addition, we manually checked all reference lists of the retrieved papers and questioned specialists to find any potential relevant research.

### 2.2. Inclusion and exclusion criteria

Studies were chosen if they met the following inclusion criteria: (1) the study design was either a randomized or crossover study; (2) participants had depressive symptoms; (3) patients were being treated using PBM (t-PBM or s-PBM); (4) the studies used widely recognized depression and sleep scales, with a primary outcome of depression and the secondary outcome of sleep.

Excluded were studies that met the following criteria: (1) conference reports, abstracts, animal trials, and replicated research; (2) the experimental group received both PBM and other treatments; (3) the objective data was either missing or could not be extracted, and the full text was unavailable after contacting the corresponding author; (4) studies in which other nondepressive outcomes interfere with the observation of depression efficacy; (5) non-Chinese and English literature.

### 2.3. Study selection

Endnote 20 was utilized to manage the search record. Two reviewers independently screened titles and abstracts for potentially qualified studies based on the inclusion criteria after deleting duplicate results. They then read the full text of potentially eligible studies to identify the final included literature. In the event of a disagreement, a third reviewer is invited to discuss and make a decision.

### 2.4. Data collection and extraction

Two reviewers (Q-pJ and S-cY) used the data extraction form to extract the required data from the included studies. The data to be extracted includes first author, time of publication, irradiance, irradiation site, wavelength, fluence, light source, treatment frequency, number of treatments, time of intervention, and outcomes. A third reviewer was asked to discuss and make a judgment call in case of disagreement.

### 2.5. Risk-of-bias assessment

Using the Cochrane assessment of the risk of bias, two reviewers (Q-pJ and S-cY) independently evaluate the bias risk. The tool divides studies into three categories according to their risk of bias: low, high, or unclear. These categories are selection bias (random sequence generation and allocation concealment), performance bias (blinding of participants and staff), detection bias (blinding of the outcome assessment), attrition bias (incomplete outcome data), reporting bias (selective reporting bias), and other biases. In the event of a dispute, a third reviewer was brought in.

### 2.6. Certainty of the evidence

The rating of recommendations is grounded in the GRADE (Grading of Recommendations Assessment, Development, and Evaluation) methodology, in which the certainty of evidence is categorized as “very low,” “low,” “medium,” or “high.” High” (49). The quality of randomized controlled trials is high, while the quality of observational studies is low. Research quality can be lowered by five factors: limitations, inconsistency, indirectness, imprecision, and publication bias (50).

### 2.7. Statistical analysis

The statistical significance threshold was set at *p <* 0.05, and the data was integrated with RevMan 5.4 and Stata 14.0. Results for this study were continuous variables, and for each effect size, the researchers calculated 95% confidence intervals (CIs). For outcomes assessed using the same scale, the data were pooled using the mean difference (MD), while for outcomes assessed using different scales, the standardized mean difference (SMD) was calculated. Statistical heterogeneity of the included studies was assessed using the chi-square test and the *I^2^* statistical measure. Low, medium, and high heterogeneity existed according to the *I^2^* statistic (25, 50, and 75%). A fixed-effects model was used when *p* > 0.1 or *I^2^* < 50%. Otherwise, a random effects model was used. Forest plots are used to display the pooled estimations. The estimates were analyzed in subgroups based on age, light source, intervention duration, wavelength, irradiance time, irradiation site, fluence, irradiance, treatment frequency, and number of treatments. Sensitivity analyses for exploring the stability of the study results were present.

### 2.8. Publication bias

When there were more than ten included studies for the outcome, possible publication bias was assessed by funnel plot, Egger’s regression, and Begg’s regression (*p* < 0.05 indicates publication bias) (51, 52). If there was a significant publication bias, a Duvaland and Tweedie trim and fill method was used to provide possible missing trials.

## 3. Results

### 3.1. Selection and inclusion of studies

**Figure 1:**
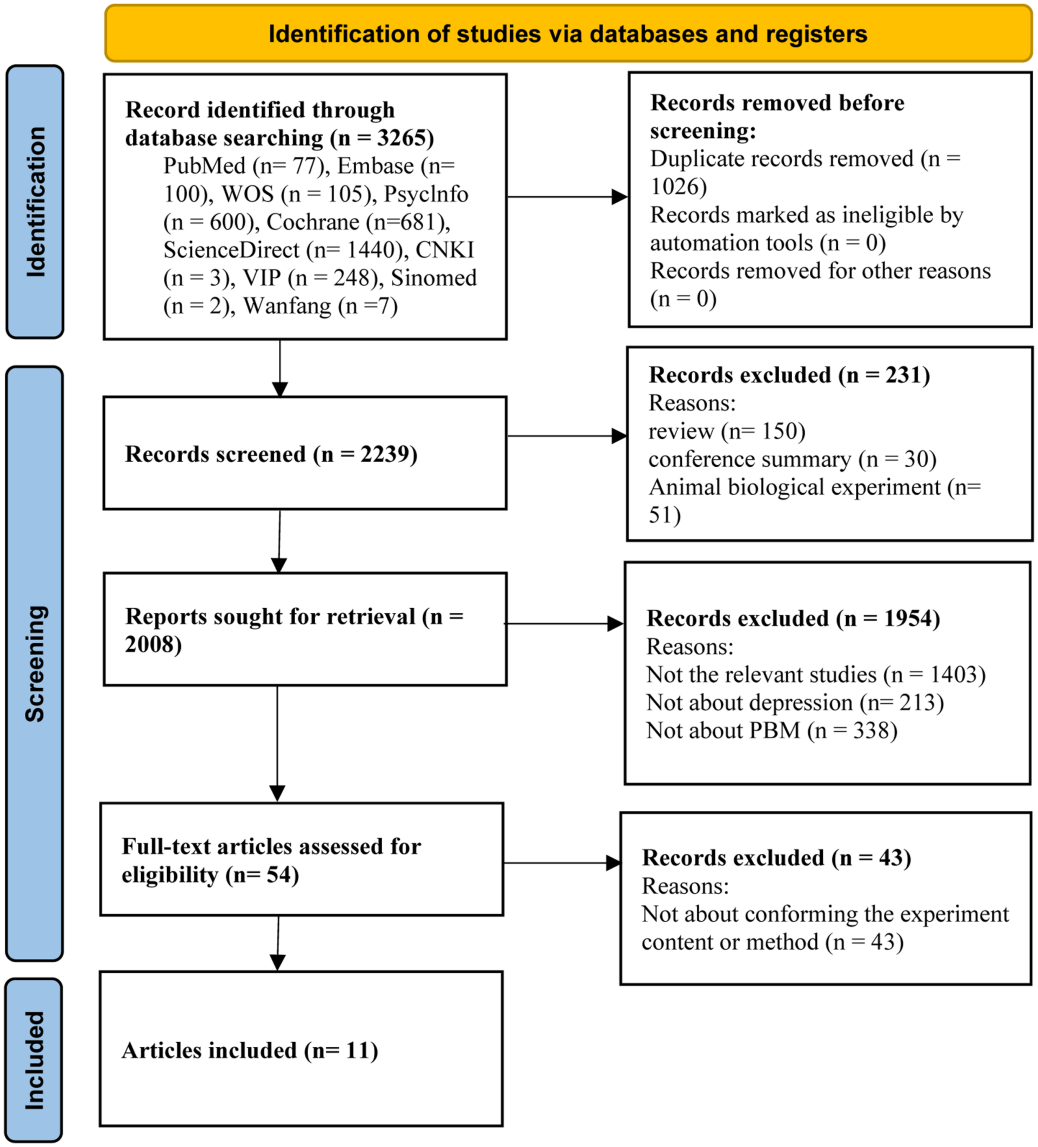
PRISMA flowchart of the literature search for the effects of photobiomodulation on depression.

An initial search of 3,265 studies was conducted through ten databases. Eleven research studies (53–63) were identified for meta-analysis through multiple screening steps, such as excluding duplicates, selecting the type of literature, systematically reviewing title and abstract contests, and reviewing the full text (Figure 1).

### 3.2. Characteristics of included studies

Table 1 presents the characterization of the included trials. A total of eleven studies met our inclusion and exclusion criteria. The study areas were mainly in the United States, Australia, and Turkey, and most participants suffered from varying degrees of depression or other mood-affecting disorders. The participants totaled 407, including 200 in the experimental group and 207 in the control group. Most of the participants were between 30 and 50 years old.

**Table 1:** Characteristics of included studies.

Study (year)	Country	Participants (n)	Mean age	Irradiation site	Irradiance	time	wavelength	treatment frequency	number of treatments	Fluence	Light source	Duration	Outcomes
Gur 2002	Türkiye	Fibromyalgia (*n* = 50)	EG:30.36 CG:28.52	tender point (s-PBM)	11.2 mW/cm^2^	3 min	904 nm	14/week	28	2 J/cm^2^	Laser	2 weeks	HDRS
Turkey 2004	Iurface	MPS (*n* = 50)	EG:32.24 CG:30.92	tender point (s-PBM)	11.2 mW/cm^2^	3 min	904 nm	14/week	28	2 J/cm^2^	Laser	2 weeks	BDI
Smith 2012	Australia	MDD (*n* = 44)	EG:40.8 CG:36.27	acupoint (s-PBM)	100 mW/cm^2^	10s	808 nm	3/week	12	1 J/cm^2^	Laser	4 weeks	HAMD
Smith 2005	Australia	MDD (*n* = 30)	EG:38.9 CG:38.1	acupoint (s-PBM)	100 mW/cm^2^	10s	808 nm	3/week	24	0.5 J/cm^2^	Laser	8 weeks	BDI
Cassano 2018	America	MDD (*n* = 20)	EG:47.3 CG:50.7	DlPFC (t-PBM)	36.2 mW/cm^2^	30 min	823 nm	2 /week	16	65.2 J/cm^2^	LED	8 weeks	HAMD
Cassano 2019	America	SD (*n* = 21)	EG:45.0 CG:50.7	DlPFC (t-PBM)	33.2 mW/cm^2^	30 min	823 nm	2 /week	16	65.2 J/cm2	LED	8 weeks	HAMD
Chen 2019	China	Sleep disorders (*n* = 25)	EG:52.69 CG:52.17	acupoint (s-PBM)	NA	15 min	808 nm	2 /week	10	540 J/cm^2^	Laser	5 weeks	HADS, PSQI
Kheradmand 2022	Iran	Schizophrenia (*n* = 32)	EG:50.24 CG:49.47	DlPFC (t-PBM)	100 mW/cm^2^	15 min	810 nm	3/week	6	144 J/cm^2^	Laser	2 weeks	PANSS
Pujante 2022	Spain	Xerostomia (*n* = 53)	EG:65.4 CG:67.8	tender point (s-PBM)	83.3 mW/cm^2^	2 min	810 nm	1/week	6	6 J/cm^2^	LED	6 weeks	HADS, PSQI
Iosifescu 2022	America	MDD (*n* = 49)	EG:37.2 CG:42.8	DlPFC (t-PBM)	54.8 mW/cm^2^	4 min	830 nm	2/week	12	65.8 J/cm^2^	LED	12 weeks	HDRS
Disner 2016	America	MDD (*n* = 32)	EG:18.72 CG:20.33	DlPFC (t-PBM)	250 mW/cm^2^	4 min	1,064 nm	2/week	4	60 J/cm^2^	LED	2 weeks	CES-D

EG, experimental group; CG, control group; NA, not available; PBM, photobiomodulation; t-PBM, transcranial PBM; s-PBM, systemic PBM; Min, minutes; s, second; LED, light-emitting diodes; HDRS, the Hamilton Depression Rate Scale; BDI, Beck depression Inventory; HAMD, Hamilton Depression Rating Scale; PSQI, the Pittsburgh Sleep Quality Index; HADS, the Hospital Anxiety and Depression Scale; CES-D, Center for Epidemiologic Studies-Depression Scale; PANSS, Positive and Negative Syndrome Scale; MPS, myofascial pain syndrome; MDD, major depressive disorder; SD, sexual dysfunction; DlPFC, dorsolateral prefrontal cortex.

Among the eleven studies, the light interventions differed in light source, Irradiation site, intervention duration, Irradiance, wavelength, fluence, treatment frequency, and number of treatments. The light source was LED in 5 studies (53, 54, 56, 57, 60, 61) and laser in 6 (55, 58, 59, 61–63). Regarding the duration of the interventions, five studies (53, 54, 56, 61, 62) were within 4 weeks, five studies (53–55, 57, 63) were within 6–8 weeks, and one study was within 9–12 weeks (60). The wavelengths of the light ranged from 808 nm to 1,064 nm. By irradiating site, PBM can be categorized into t-PBM and s-PBM, where five studies (53, 54, 56, 60, 61) treated the brain directly (t-PBM) exposing the DIPFC, three (57–59) treated at a tender acupoint, and three in different acupoint (55, 62, 63). Based on treatment frequency, six (53–57, 60) studies treated <3/week and five studies ≥3/week (58, 59, 61–63). Based on the number of treatments, six (55–57, 60–62) studies treated ≤15 times and five studies >15 (53, 54, 58, 59, 63). Regarding the fluence, two studies were within ≤1 J/cm^2^ (62, 63), three studies within 1–10 J/cm^2^ (57–59), four studies within 10–100 J/cm^2^ (53, 54, 56, 60), and two study within 100–1,000 J/cm^2^ (55, 61). For the irradiance, four studies were within ≤50 mW/cm^2^ (53, 54, 58, 59), five were within 50–100 mW/cm^2^ (57, 60–63), and one was 250 mW/cm^2^ (56).

### 3.3. Risk of bias in studies

**Figure 2:**
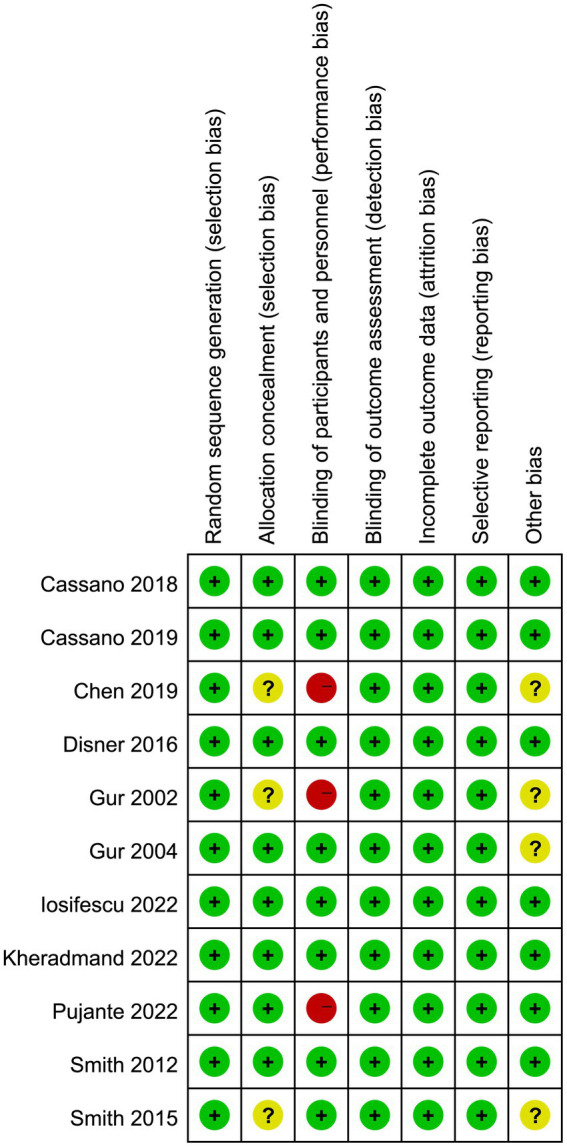
Risk of bias summery.

**Figure 3:**
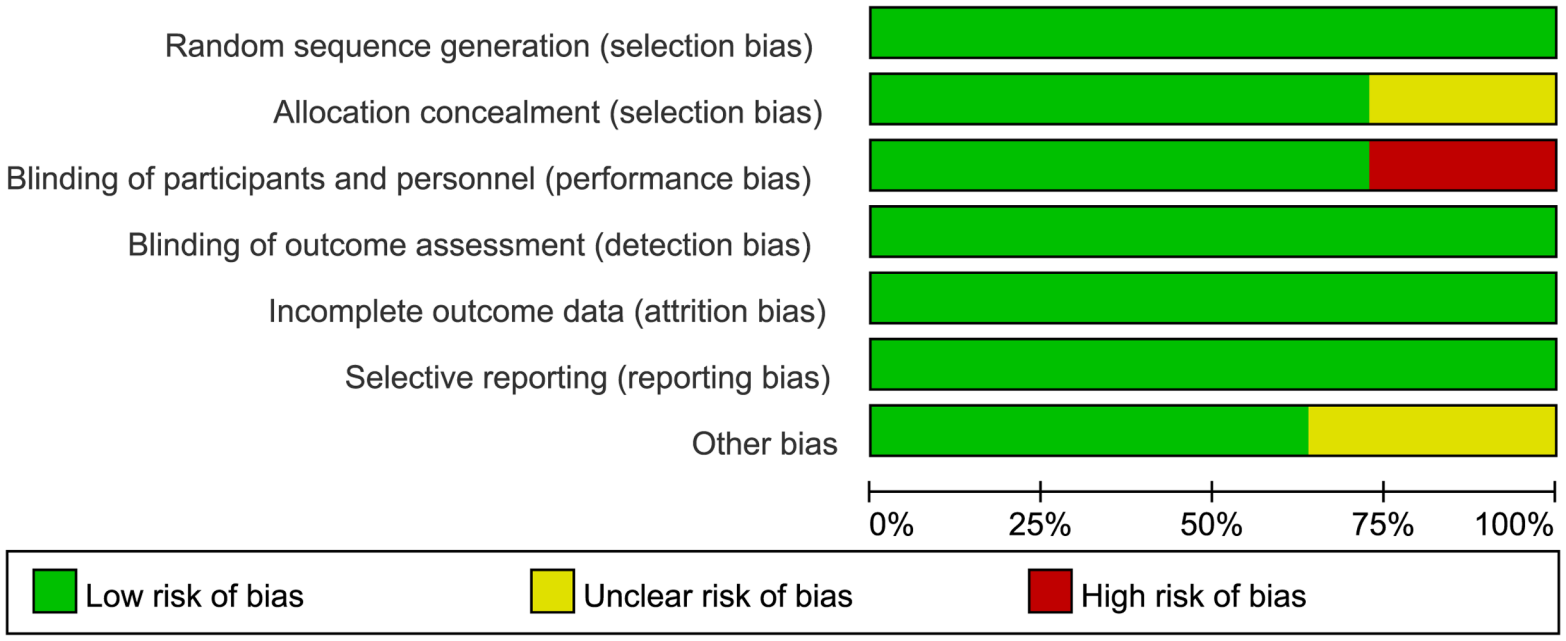
Risk of bias graph.

The risk of bias graph for each inclusion study is shown in Figure 2, and the percentages for each study are shown in Figure 3. Of the eleven included studies, all mentioned random sequence generation. Regarding allocation concealment, three studies were assessed as having some problems because details of assignment concealment were not mentioned, and eight studies reported details of allocation concealment. Nine studies mentioned blinding in their studies. Although the studies used different types of interventions, participants and staff were not informed whether they were in the experimental or control group. Three studies did not mention blinding of participants and staff. In addition, incomplete outcome data and selective reporting were not mentioned in any of the studies. Regarding other biases, seven studies were considered to be at low risk, and the remaining four were considered to have some problems.

### 3.4. Results of the meta-analysis

#### 3.4.1. Depression

**Figure 4:**
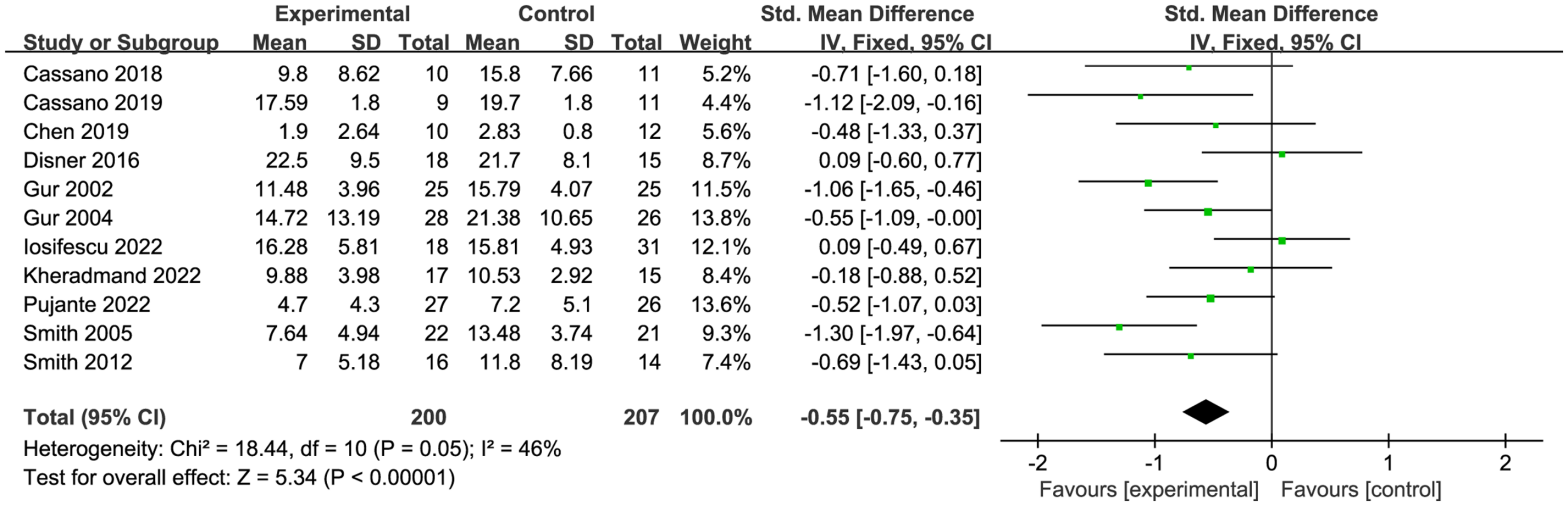
Forest plot of depression outcome.

**Figure 5:**
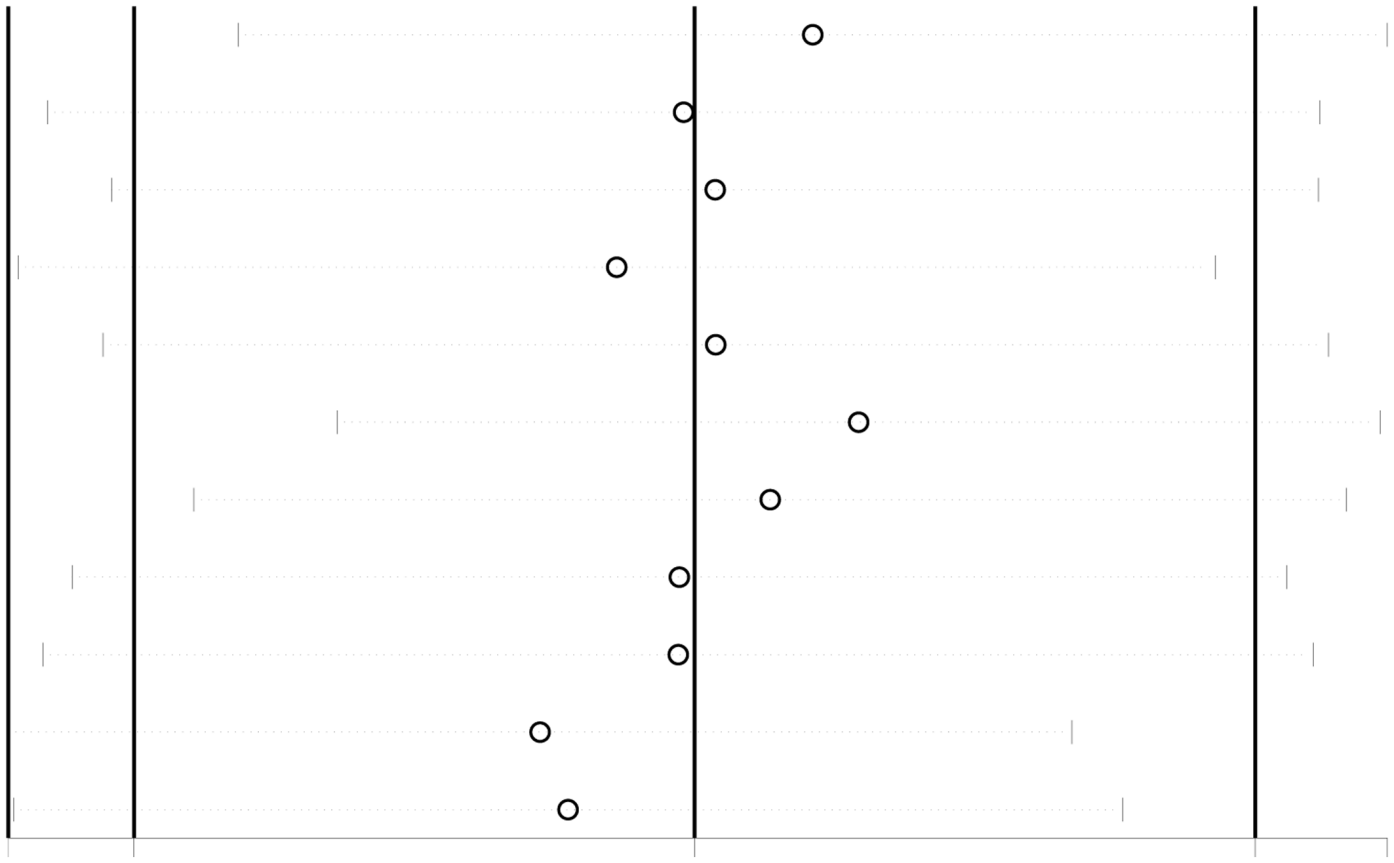
Sensitivity analysis of depression.

A total of eleven trials (53–63) reported on 407 patients with depression. Meta-analysis of fixed effects showed that PBM improved patients’ depressive symptoms compared with controls (SMD = −0.55, 95% CI [−0.75, −0.35], *I^2^* = 46%; Figure 4). Sensitivity analyses showed that exclusion of each study did not affect the stability of our trial (Figure 5).

##### 3.4.1.1. Subgroup analysis

Subgroups were analyzed as shown in Table 2 (supplementing the forest plot in Appendix 4). The light was broadly categorized according to where it was applied in the body: transcranial PBM (t-PBM) and systemic PBM (s-PBM). In terms of improvement in depression, s-PBM was superior to t-PBM. A subgroup analysis of the efficacy of t-PBM on depression showed that adults under 40 years of age showed more significant improvement in depression than adults over 40 years of age; the light source was more effective with LED than with a laser; and an irradiance time of 30 min provided the best improvement in depression; Light with a wavelength of 823 nm is best; fluence of 10–100 J/cm^2^ is better than 100–1,000 J/cm^2^ for depression; irradiance ≤50 mW/cm^2^ is best; treatment frequency < 3/week is best; number of treatments >15 times is the best.

**Table 2:** Subgroup analyses of depression outcome (t-PBM and s-PBM).

Outcome or subgroup	Number of studies	Patients (E/C)	Overall effect size (95% CI) *p*		Heterogeneity *I^2^ p*	
t-PBM						
Overall	5	72/83	−0.21 [−0.54, 0.11]	0.20	38%	0.17
**Age**						
≤40	2	36/37	−0.09 [−0.36, 0.56]	0.70	0%	1.00
>40	3	36/37	−0.56 [−1.04, −0.09]	**0.02**	22%	0.28
Light source						
LED	4	55/68	−0.22 [−0.59, 0.14]	0.23	53%	0.09
Laser	1	17/15	−0.18 [−0.88, −0.52]	0.61	-	-
Irradiance time						
≤5 min	2	36/46	0.09 [−0.36, 0.53]	0.70	0%	1.00
15 min	1	17/15	−0.18 [−0.88, 0.52]	0.61	-	-
30 min	2	19/22	−0.90 [−1.55, −0.25]	**0.007**	0%	0.54
Wavelength						
810 nm	1	17/15	−0.18 [−0.88, 0.52]	0.61	-	-
823 nm	2	19/22	−0.90 [−1.55, −0.25]	**0.007**	0%	0.54
830 nm	1	18/31	0.09 [−0.49, 0.67]	0.77	-	-
1,064 nm	1	18/15	−0.09 [−0.60, 0.77]	0.80	-	-
Fluence						
10–100 J/cm^2^	4	55/68	−0.22 [−0.59, 0.14]	0.23	53%	0.09
100–1,000 J/cm^2^	1	17/15	−0.18 [−0.88, 0.52]	0.61	-	-
Irradiance						
≤50 mW/cm^2^	3	37/53	−0.35 [−0.78, 0.09]	0.12	62%	0.07
50–100 mW/cm^2^	2	35/30	−0.04 [−0.53, 0.44]	0.86	0%	0.59
Treatment frequency						
< 3/week	4	55/68	−0.22 [−0.59, 0.14]	0.23	53%	0.09
≥ 3/week	1	17/15	−0.18 [−0.88, −0.52]	0.61	-	-
Number of treatments						
≤ 15	3	53/61	0.01 [−0.36, 0.38]	0.96	0%	0.82
> 15	2	19/22	−0.90 [−1.55, −0.25]	**0.007**	0%	0.54
s-PBM						
Overall	6	128/124	−0.76 [−1.02, −0.50]	**<0.00001**	6%	0.38
Age						
≤40	4	91/86	−0.88 [−1.19, −0.57]	**<0.00001**	17%	0.31
>40	2	37/38	−0.51 [−0.97, −0.05]	**0.03**	0%	0.93
Irradiation site						
tender point	3	80/77	−0.69 [−1.01, −0.37]	**<0.0001**	4%	0.35
acupoint	3	47/48	−0.89 [−1.32, −0.46]	**<0.0001**	25%	0.26
Light source						
LED	1	27/26	−0.52 [−1.07, 0.03]	0.06	-	-
Laser	5	101/98	−0.83 [−1.12, −0.54]	**<0.0001**	8%	0.36
Irradiance time						
≤5 min	5	118/112	−0.79 [−1.06, −0.52]	**<0.0001**	17%	0.31
15 min	1	10/12	−0.48 [−1.33, 0.37]	0.27	-	-
Wavelength						
808 nm	3	48/47	−0.89 [−1.32, −0.46]	**<0.0001**	25%	0.26
810 nm	1	27/26	−0.52 [−1.07, 0.03]	0.06	-	-
904 nm	2	53/51	−0.78 [−1.18, −0.38]	**0.0001**	35%	0.21
Fluence						
≤ 1 J/cm^2^	2	38/35	−1.03 [−1.53, 0.54]	**<0.0001**	31%	0.23
1–10 J/cm^2^	3	80/77	−0.69 [−1.02, −0.36]	**<0.0001**	4%	0.35
Irradiance						
≤ 50 mW/cm^2^	2	53/51	−0.78 [−1.18, −0.38]	**0.0001**	35%	0.21
50–100 mW/cm^2^	3	65/61	−0.80 [−1.17, −0.44]	**<0.0001**	39%	0.19
Treatment frequency						
< 3/week	2	37/38	−0.51 [−0.97, −0.05]	**0.03**	0%	0.93
≥ 3/week	4	91/86	−0.88 [−1.19, −0.57]	**<0.00001**	17%	0.31
Number of treatments						
≤ 15	3	53/52	−0.56 [−0.95, −0.17]	**0.005**	0%	0.92
> 15	3	75/72	−0.92 [−1.26, −0.58]	**<0.00001**	40%	0.19

LED, light-emitting diodes; PBM, photobiomodulation; t-PBM, transcranial PBM; s-PBM, systemic PBM; Min, minute; nm, nanometer. Bold value means *p* < 0.05.

Subgroup analyses of the efficacy of s-PBM on depression showed that s-PBM improved depression better in adults under 40 years old; irradiation site was more effective at the acupoint than at the tender point; light source was more effective at the laser; and irradiance time was best when it was ≤5 min; Wavelength of 808 nm is best for depression; fluence at ≤1 J/cm^2^ best improves depression; irradiance of 50–100 mW/cm^2^ is best; treatment frequency ≥ 3/week is best; number of treatments >15 times is best.

##### 3.4.1.2. Publication bias

**Figure 6:**
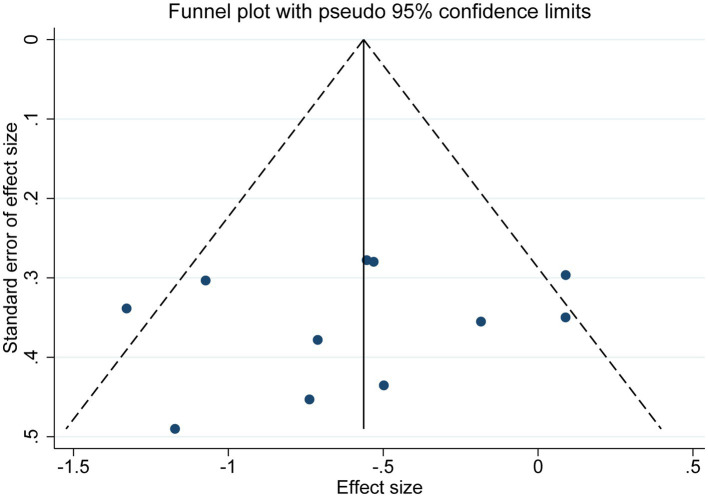
The funnel graph of depression.

As shown in Figure 6, publication bias was examined by funnel plot and Egger’s and Begg’s tests. The funnel plot showed basic symmetry with *p* = 0.547 in Egger’s test and *p* = 0.533 in Begg’s test, indicating no publication bias (*p* > 0.05).

#### 3.4.2. Sleep

**Figure 7:**
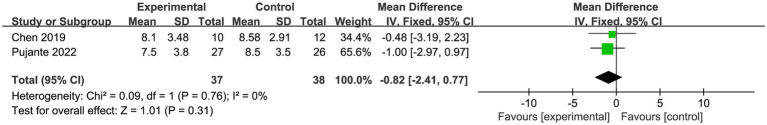
Forest plot of sleep outcome.

A total of two trials (55, 57) with 75 patients reported sleep. Meta-analysis of fixed effects showed no statistically significant difference in patient sleep in the PBM (s-PBM) group compared to the control group (SMD = −0.82, 95% CI [−2.41, 0.77], *I^2^* = 0%; *p* > 0.05; Figure 7).

### 3.5. Certainty of the evidence

The GRADE results are presented in Supplementary Appendix 3. The level of evidence certainty was rated as “moderate” for depression and ‘very low’ for sleep. The leading causes of the sample size of the included study were too small, and the confidence interval was too broad.

## 4. Discussion

### 4.1. The effect of PBM on depression and sleep

A total of eleven randomized controlled trials was included in this meta-analysis, and the combined data suggest that PBM may be an efficacious depression treatment. Neurophysiological studies have confirmed that important pathological causes of depression include neuroinflammation, reduced levels of monoamine neurotransmitters, and mitochondrial dysfunction (64–67). PBM has been increased for the treatment of depression, and it stimulates neuronal processes in the brain as well as CCO, the excitation of which leads to an increase in neuronal ATP synthesis (68). These low-level lasers can modulate nitric oxide (NO) release (68, 69) thereby increasing brain oxygenation and blood flow. In addition to this, PBM modulates neurotransmitter metabolism, reduces neuroinflammation and oxidative stress, and thus acts as a neuroprotective agent (70). Many clinical trials have shown that PBM can be effective in alleviating depressive symptoms. Schiffer et al. (31) used the t-PBM to treat MDD patients suffering from anxiety disorders and traumatic stress disorders, and the research showed a significant improvement in depression and anxiety in the second and fourth weeks.

Regarding the sleep aspect, the results of this study showed no statistically significant effect of PBM on sleep. Therefore, more randomized controlled trials may be needed to determine the role of PBM in sleep. The current study suggests that PBM may improve PSQI scores and sleep quality by modulating the circadian rhythm of cortisol levels (71, 72). Several studies have also reported that PBM improves sleep quality in patients, but the exact mechanism is still under investigation (73–75).

### 4.2. The prognosis of PBM for the treatment of depression

According to subgroup analyses, t-PBM and s-PBM showed opposite effects on the improvement of depression in terms of age. t-PBM showed better effects in patients aged 40 years or older; the opposite was true for s-PBM. Previous studies have shown that t-PBM affects prefrontal cortical CCO, which plays a key role in mitochondrial respiration (76). Whereas changes in CCO increase with age, older adults may benefit more from t-PBM treatment, which is consistent with our findings (77). Interestingly, s-PBM appeared to be more beneficial for younger participants in our study. This may be because fewer studies included middle-aged and older patients. Few studies have focused on the effects of PBM on depressed patients of different ages, so more research is needed to validate this finding.

PBM has started experimenting with different light sources (laser and LED). Our findings suggest that s-PBM has more dramatic improvements in depression with laser as the light source. This may be because lasers have greater penetration and irradiance than LEDs (78). t-PBM was best with LEDs as a light source, but the effect was insignificant. Although it is debated whether lasers are superior to LEDs, there is evidence that LEDs are comparable to lasers (15) and are safer and less costly (79, 80). t-PBMs are typically available at wavelengths in the 600–1,164 nm region, and wavelengths in this range can stimulate CCO activity more (81). Our results validate this theory, and subgroup analysis showed that PBM at 823 nm and 808 nm wavelengths showed the best improvement in depression. Several studies have confirmed that t-PBM at 808 nm is superior to t-PBM at other wavelengths regarding brain tissue penetration (82). However, according to Mie’s theory, light at 1064 nm scatters more, penetrates more, and may excite more CCOs than at 600–900 nm (82, 83). Therefore, more research is needed to explore the PBM wavelengths most suitable for treating depression.

It has been indicated that depression is associated with the metabolism of the ventral-lateral and dorsal-medial prefrontal cortex (84). In our included study, t-PBM could irradiate the DLPFC region in F3 F4 on the patient’s scalp. This suggests that DLPF may be associated with mechanisms that modulate antidepressant effects. At the same time, s-PBM may have systemic anti-inflammatory, antioxidant, and pro-metabolic effects (85, 86). In our study, s-PBM irradiated patients’ acupoints and tender points. A case–control trial mentioned the possible antidepressant effect of s-PBM on the back and thighs of patients with low back pain combined with depression (41). It is difficult to establish a causal relationship when pain and depression coexist because chronic low back pain may lead to depression, and somatic pain is a common symptom of depression (87). In this trial, five patients with LBP comorbid with depression were treated with physical therapy and s-PBM and retrospectively matched with five control patients who received physical therapy alone. Depression scores decreased significantly more in the s-PBM group than in the control group after treatment. This case–control study demonstrated that s-PBM possesses antidepressant effects. It has been proposed that the systemic effects of s-PBM through the mechanism of blood irradiation can also ultimately be neuroprotective in the brain (88). The brain will benefit from PBM’s ability to down-regulate pro-inflammatory factors and to increase anti-inflammatory cytokines irrespective of the site of treatment (88).

### 4.3. The dose-dependent effects of PBM

Longer treatment times and higher irradiance PBMs may lead to longer-lasting results (74). In our study, t-PBM with irradiance time of 30 min, treatment frequency < 3/week, and number of treatments >15 times showed the best improvement in depressive symptoms, which is consistent with the above findings; however, subgroup analysis of s-PBM showed that s-PBM was best at irradiance time ≤ 5 min, treatment frequency ≥ 3/week, and number of treatments >15 times. This may be related to the biphasic dose response of PBM, i.e., PBM regimens based on excessive energy density (J/cm^2^), excessive irradiance (mW/cm^2^), or excessive number of repetitions with longer duration are significantly less effective than t-PBM regimens based on lower doses (89, 90). The biphasic response has been demonstrated several times in PBM studies (91, 92). Fluence-based subgroup analyses suggest that t-PBM (10–100 J/cm^2^) and s-PBM (1–10 J/cm^2^) may have higher antidepressant effects. This may be because most of the photons of t-PBM are absorbed or dispersed by the scalp, skull, and cerebrospinal fluid, and only a small portion may reach the neural tissues (93). Thus, a greater fluence is required to achieve an antidepressant effect. t-PBM at 0–50 mW/cm^2^ possesses a more substantial antidepressant effect than t-PBM at 50–100 mW/cm^2^, which the biphasic response of the PBM can also explain. The biphasic response of PBM has been verified in various *in vitro* and *in vivo* studies and animal models (94). This phenomenon suggests that excessive irradiance and excessively long irradiation times may have inhibitory effects. Various studies have shown that low to moderate doses of PBM promote cell growth, whereas high intensities counteract the beneficial effects of PBM on various cell types (90).

In summary, the dose of t-PBM can be considered: irradiance time of 30 min, fluence of 10–100 J/cm^2^, irradiance ≤50 mW/cm^2^, treatment frequency < 3/week, number of treatments >15 times; Dose of s-PBM can be considered: irradiance time ≤ 5 min, fluence of ≤1 J/cm^2^, irradiance of 50–100 mW/cm^2^, treatment frequency ≥ 3/week, number of treatments >15 times. However, due to the limited number of studies, the optimal regimen and potential influences of PBM for depression have not been determined, and more rigorously designed randomized controlled trials are needed to address these issues.

### 4.4. Limitations of this study

There are some potential limitations of our study. Firstly, the small sample size of the included studies may affect the accuracy of the results. Second, some of the included studies did not explicitly report the blinding method, which may present a risk of bias. Finally, significant heterogeneity may result from the different locations, doses, and wavelengths of PBM irradiation in the studies we included.

## 5. Conclusion

In conclusion, we conclude that PBM effectively improves depression in patients. Although it is not possible to determine the specific parameters for obtaining the best results, the following can be considered in this study: t-PBM wavelength selection 823 nm, fluence selection 10–100 J/cm^2^, irradiance selection ≤ 50 mW/cm^2^, irradiance time selection 30 min, treatment frequency < 3/week, number of treatments >15 times; s-PBM wavelength selection 808 nm, fluence selection ≤1 J/cm^2^, irradiance selection 50–100 mW/cm^2^, irradiance time ≤ 5 min, treatment frequency ≥ 3/week, number of treatments >15 times. However, the current number of studies is low, and further studies are needed to extend the current analysis. More rigorous randomized controlled trials are used to understand PBM’s physiological mechanisms and beneficial effects on depression.

## Data availability statement

The original contributions presented in the study are included in the article/Supplementary material, further inquiries can be directed to the corresponding authors.

## Author contributions

QJ: Writing – original draft, Writing – review & editing. SY: Writing – review & editing. JD: Writing – review & editing. XZ: Writing – review & editing. ZL: Writing – original draft. TZ: Writing – review & editing. ZW: Writing – review & editing. WW: Writing – review & editing. HL: Writing – review & editing. SL: Writing – review & editing. SA: Writing – original draft, Writing – review & editing.

## References

[1] Mirzaei M Yasini Ardekani SM Mirzaei M Dehghani A. Prevalence of depression, anxiety and stress among adult population: results of Yazd health study. Iran J Psychiatry. (2019) 14:137–46. doi: 10.18502/ijps.v14i2.993 31440295 PMC6702282

[2] Global, Regional, and National Incidence. Prevalence, and years lived with disability for 354 diseases and injuries for 195 countries and territories, 1990-2017: a systematic analysis for the global burden of disease study 2017. Lancet. (2018) 392:1789–858. doi: 10.1016/s0140-6736(18)32279-7, PMID: 30496104 PMC6227754

[3] Cohen BE Edmondson D Kronish IM. State of the art review: depression, stress, anxiety, and cardiovascular disease. Am J Hypertens. (2015) 28:1295–302. doi: 10.1093/ajh/hpv047, PMID: 25911639 PMC4612342

[4] Strine TW Mokdad AH Balluz LS Gonzalez O Crider R Berry JT . Depression and anxiety in the United States: findings from the 2006 behavioral risk factor surveillance system. Psychiatr Serv. (2008) 59:1383–90. doi: 10.1176/ps.2008.59.12.1383, PMID: 19033164

[5] Li H Chen Z Gong Q Jia Z. Voxel-wise Meta-analysis of task-related brain activation abnormalities in major depressive disorder with suicide behavior. Brain Imaging Behav. (2020) 14:1298–308. doi: 10.1007/s11682-019-00045-3, PMID: 30790165

[6] Otte C Gold SM Penninx BW Pariante CM Etkin A Fava M . Major depressive disorder. Nat Rev Dis Primers. (2016) 2:16065. doi: 10.1038/nrdp.2016.65 27629598

[7] Li Y Zhang X Winkelman JW Redline S Hu FB Stampfer M . Association between insomnia symptoms and mortality: a prospective study of U.S. Men Circulation. (2014) 129:737–46. doi: 10.1161/circulationaha.113.004500, PMID: 24226807 PMC3987964

[8] Mc Carthy CE. Sleep disturbance, sleep disorders and co-morbidities in the Care of the Older Person. Med Sci (Basel). (2021) 9:31. doi: 10.3390/medsci9020031, PMID: 34063838 PMC8162526

[9] Rod NH Kumari M Lange T Kivimäki M Shipley M Ferrie J. The joint effect of sleep duration and disturbed sleep on cause-specific mortality: results from the Whitehall ii cohort study. PLoS One. (2014) 9:e91965. doi: 10.1371/journal.pone.0091965 24699341 PMC3974730

[10] Locke AB Kirst N Shultz CG. Diagnosis and management of generalized anxiety disorder and panic disorder in adults. Am Fam Physician. (2015) 91:617–24. 25955736

[11] Guideline Development Panel for the Treatment of Depressive Disorders. Summary of the clinical practice guideline for the treatment of depression across three age cohorts. Am Psychol. (2022) 77:770–80. doi: 10.1037/amp0000904 34843274

[12] Sowa-Kućma M Pańczyszyn-Trzewik P Misztak P Jaeschke RR Sendek K Styczeń K . Vortioxetine: a review of the pharmacology and clinical profile of the novel antidepressant. Pharmacol Rep. (2017) 69:595–601. doi: 10.1016/j.pharep.2017.01.030, PMID: 28499187

[13] Chung H Dai T Sharma SK Huang YY Carroll JD Hamblin MR. The nuts and bolts of low-level laser (light) therapy. Ann Biomed Eng. (2012) 40:516–33. doi: 10.1007/s10439-011-0454-7, PMID: 22045511 PMC3288797

[14] Gonzalez-Lima F Barrett DW. Augmentation of cognitive brain functions with transcranial lasers. Front Syst Neurosci. (2014) 8:36. doi: 10.3389/fnsys.2014.00036 24672439 PMC3953713

[15] Hamblin MR. Shining light on the head: photobiomodulation for brain disorders. BBA Clin. (2016) 6:113–24. doi: 10.1016/j.bbacli.2016.09.002, PMID: 27752476 PMC5066074

[16] Wong-Riley MT Liang HL Eells JT Chance B Henry MM Buchmann E . Photobiomodulation directly benefits primary neurons functionally inactivated by toxins: role of cytochrome C oxidase. J Biol Chem. (2005) 280:4761–71. doi: 10.1074/jbc.M409650200, PMID: 15557336

[17] Salehpour F Khademi M Bragin DE DiDuro JO. Photobiomodulation therapy and the Glymphatic system: promising applications for augmenting the brain lymphatic drainage system. Int J Mol Sci. (2022) 23:2975. doi: 10.3390/ijms23062975, PMID: 35328396 PMC8950470

[18] Mochizuki-Oda N Kataoka Y Cui Y Yamada H Heya M Awazu K. Effects of near-infra-red laser irradiation on adenosine triphosphate and adenosine diphosphate contents of rat brain tissue. Neurosci Lett. (2002) 323:207–10. doi: 10.1016/s0304-3940(02)00159-3, PMID: 11959421

[19] Oron A Oron U Streeter J de Taboada L Alexandrovich A Trembovler V . Low-level laser therapy applied Transcranially to mice following traumatic brain injury significantly reduces long-term neurological deficits. J Neurotrauma. (2007) 24:651–6. doi: 10.1089/neu.2006.0198, PMID: 17439348

[20] Yu W Naim JO McGowan M Ippolito K Lanzafame RJ. Photomodulation of oxidative metabolism and Electron chain enzymes in rat liver mitochondria. Photochem Photobiol. (1997) 66:866–71. doi: 10.1111/j.1751-1097.1997.tb03239.x 9421973

[21] Tripathi A Scaini G Barichello T Quevedo J Pillai A. Mitophagy in depression: pathophysiology and treatment targets. Mitochondrion. (2021) 61:1–10. doi: 10.1016/j.mito.2021.08.016, PMID: 34478906 PMC8962570

[22] Andreazza AC Andersen ML Alvarenga TA De-Oliveira MR Armani F Ruiz FS . Impairment of the mitochondrial electron transport chain due to sleep deprivation in mice. J Psychiatr Res. (2010) 44:775–80. doi: 10.1016/j.jpsychires.2010.01.015 20176368

[23] Mutisya EM Bowling AC Beal MF. Cortical cytochrome oxidase activity is reduced in Alzheimer's disease. J Neurochem. (1994) 63:2179–84. doi: 10.1046/j.1471-4159.1994.63062179.x 7964738

[24] Parker WD Jr. Cytochrome oxidase deficiency in Alzheimer's disease. Ann N Y Acad Sci. (1991) 640:59–64. doi: 10.1111/j.1749-6632.1991.tb00191.x 1663716

[25] Yang F Qi X Gao Z Yang X Zheng X Duan C . Homocysteine injures vascular endothelial cells by inhibiting mitochondrial activity. Exp Ther Med. (2016) 12:2247–52. doi: 10.3892/etm.2016.3564, PMID: 27698720 PMC5038564

[26] de Freitas LF Hamblin MR. Proposed mechanisms of Photobiomodulation or low-level light therapy. IEEE J Sel Top Quantum Electron. (2016) 22:348–64. doi: 10.1109/jstqe.2016.2561201, PMID: 28070154 PMC5215870

[27] Lapchak PA De Taboada L. Transcranial near infrared laser treatment (Nilt) increases cortical Adenosine-5′-triphosphate (Atp) content following embolic strokes in rabbits. Brain Res. (2010) 1306:100–5. doi: 10.1016/j.brainres.2009.10.022, PMID: 19837048

[28] Nizamutdinov D Qi X Berman MH Dougal G Dayawansa S Wu E . Transcranial near infrared light stimulations improve cognition in patients with dementia. Aging Dis. (2021) 12:954–63. doi: 10.14336/ad.2021.0229, PMID: 34221541 PMC8219492

[29] Rojas JC Gonzalez-Lima F. Neurological and psychological applications of transcranial lasers and Leds. Biochem Pharmacol. (2013) 86:447–57. doi: 10.1016/j.bcp.2013.06.012, PMID: 23806754

[30] Barrett DW Gonzalez-Lima F. Transcranial infrared laser stimulation produces beneficial cognitive and emotional effects in humans. Neuroscience. (2013) 230:13–23. doi: 10.1016/j.neuroscience.2012.11.016 23200785

[31] Schiffer F Johnston AL Ravichandran C Polcari A Teicher MH Webb RH . Psychological benefits 2 and 4 weeks after a single treatment with near infrared light to the forehead: a pilot study of 10 patients with major depression and anxiety. Behav Brain Funct. (2009) 5:46. doi: 10.1186/1744-9081-5-46, PMID: 19995444 PMC2796659

[32] Moro C Valverde A Dole M Hoh Kam J Hamilton C Liebert A . The effect of photobiomodulation on the brain during wakefulness and sleep. Front Neurosci. (2022) 16:942536. doi: 10.3389/fnins.2022.942536, PMID: 35968381 PMC9366035

[33] Byrnes KR Waynant RW Ilev IK Wu X Barna L Smith K . Light promotes regeneration and functional recovery and alters the immune response after spinal cord injury. Lasers Surg Med. (2005) 36:171–85. doi: 10.1002/lsm.20143, PMID: 15704098

[34] Muili KA Gopalakrishnan S Meyer SL Eells JT Lyons JA. Amelioration of experimental autoimmune encephalomyelitis in C57bl/6 mice by photobiomodulation induced by 670 nm light. PLoS One. (2012) 7:e30655. doi: 10.1371/journal.pone.0030655, PMID: 22292010 PMC3265499

[35] Saliba A Du Y Liu H Patel S Roberts R Berkowitz BA . Photobiomodulation mitigates diabetes-induced retinopathy by direct and indirect mechanisms: evidence from intervention studies in pigmented mice. PLoS One. (2015) 10:e0139003. doi: 10.1371/journal.pone.0139003 26426815 PMC4591336

[36] Farfara D Tuby H Trudler D Doron-Mandel E Maltz L Vassar RJ . Low-level laser therapy ameliorates disease progression in a mouse model of Alzheimer's disease. J Mol Neurosci. (2015) 55:430–6. doi: 10.1007/s12031-014-0354-z, PMID: 24994540

[37] Al Amir Dache Z Otandault A Tanos R Pastor B Meddeb R Sanchez C . Blood contains circulating cell-free respiratory competent mitochondria. FASEB J. (2020) 34:3616–30. doi: 10.1096/fj.201901917RR, PMID: 31957088

[38] Johnstone DM el Massri N Moro C Spana S Wang XS Torres N . Indirect application of near infrared light induces neuroprotection in a mouse model of parkinsonism – an Abscopal neuroprotective effect. Neuroscience. (2014) 274:93–101. doi: 10.1016/j.neuroscience.2014.05.023 24857852

[39] Hamblin MR. Photobiomodulation and the brain – has the light dawned? Biochemist. (2016) 38:24–8. doi: 10.1042/bio03806024

[40] Cassano P Cusin C Mischoulon D Hamblin MR De Taboada L Pisoni A . Near-infrared transcranial radiation for major depressive disorder: proof of concept study. Psychiatry J. (2015) 2015:352979:1–8. doi: 10.1155/2015/352979, PMID: 26356811 PMC4556873

[41] Gabel CP Petrie SR Mischoulon D Hamblin MR Yeung A Sangermano L . A case control series for the effect of photobiomodulation in patients with low back pain and concurrent depression. Laser Ther. (2018) 27:167–73. doi: 10.5978/islsm.27_18-OR-18, PMID: 32158062 PMC7034249

[42] Zalewska-Kaszubska J Obzejta D. Use of low-energy laser as adjunct treatment of alcohol addiction. Lasers Med Sci. (2004) 19:100–4. doi: 10.1007/s10103-004-0307-9, PMID: 15674998

[43] Cardoso FS De Souza Oliveira Tavares C BHS A Mansur F RÁB L-M Gomes Da Silva S. Improved spatial memory and neuroinflammatory profile changes in aged rats submitted to photobiomodulation therapy. Cell Mol Neurobiol. (2022) 42:1875–86. doi: 10.1007/s10571-021-01069-4, PMID: 33704604 PMC11421705

[44] Rhie SJ Jung EY Shim I. The role of neuroinflammation on pathogenesis of affective disorders. J Exerc Rehabil. (2020) 16:2–9. doi: 10.12965/jer.2040016.008, PMID: 32161729 PMC7056473

[45] Zhang D Shen Q Wu X Xing D. Photobiomodulation therapy ameliorates glutamatergic dysfunction in mice with chronic unpredictable mild stress-induced depression. Oxidative Med Cell Longev. (2021) 2021:6678276–20. doi: 10.1155/2021/6678276, PMID: 33859781 PMC8024102

[46] Cassano P Petrie SR Hamblin MR Henderson TA Iosifescu DV. Review of transcranial photobiomodulation for major depressive disorder: targeting brain metabolism, inflammation, oxidative stress, and neurogenesis. Neurophotonics. (2016) 3:031404. doi: 10.1117/1.NPh.3.3.031404, PMID: 26989758 PMC4777909

[47] Cho Y Tural U Iosifescu DV. Efficacy of transcranial photobiomodulation on depressive symptoms: a Meta-analysis. Photobiomodul Photomed Laser Surg. (2023) 41:460–6. doi: 10.1089/photob.2023.0041, PMID: 37651208 PMC10518694

[48] Page MJ McKenzie JE Bossuyt PM Boutron I Hoffmann TC Mulrow CD . The Prisma 2020 statement: an updated guideline for reporting systematic reviews. BMJ. (2021) 372:n71. doi: 10.1136/bmj.n71, PMID: 33782057 PMC8005924

[49] Balshem H Helfand M Schünemann HJ Oxman AD Kunz R Brozek J . Grade guidelines: 3. Rating the quality of evidence. J Clin Epidemiol. (2011) 64:401–6. doi: 10.1016/j.jclinepi.2010.07.015 21208779

[50] Guyatt GH Oxman AD Schünemann HJ Tugwell P Knottnerus A. Grade guidelines: a new series of articles in the journal of clinical epidemiology. J Clin Epidemiol. (2011) 64:380–2. doi: 10.1016/j.jclinepi.2010.09.011 21185693

[51] Begg CB Mazumdar M. Operating characteristics of a rank correlation test for publication Bias. Biometrics. (1994) 50:1088–101. doi: 10.2307/2533446, PMID: 7786990

[52] Irwig L Macaskill P Berry G Glasziou P. Bias in meta-analysis detected by a simple, graphical test. Graphical test is itself biased. BMJ. (1998) 316:469. doi: 10.1136/bmj.316.7129.469 PMC2665595 9492687

[53] Cassano P Dording C Thomas G Foster S Yeung A Uchida M . Effects of transcranial photobiomodulation with near-infrared light on sexual dysfunction. Lasers Surg Med. (2019) 51:127–35. doi: 10.1002/lsm.23011, PMID: 30221776 PMC6382556

[54] Cassano P Petrie SR Mischoulon D Cusin C Katnani H Yeung A . Transcranial photobiomodulation for the treatment of major depressive disorder. The Elated-2 pilot trial. Photomed Laser Surg. (2018) 36:634–46. doi: 10.1089/pho.2018.4490, PMID: 30346890 PMC7864111

[55] Chen CK Lin YC Cheng JW Pei YC Liu GH Chen YL . Effectiveness of laser acupuncture in alleviating chronic insomnia: a single-blinded randomized controlled trial. Evid Based Complement Alternat Med. (2019) 2019:8136967–9. doi: 10.1155/2019/8136967, PMID: 31312225 PMC6595393

[56] Disner SG Beevers CG Gonzalez-Lima F. Transcranial laser stimulation as neuroenhancement for attention bias modification in adults with elevated depression symptoms. Brain Stimul. (2016) 9:780–7. doi: 10.1016/j.brs.2016.05.009 27267860 PMC5007141

[57] Ferrandez-Pujante A Pons-Fuster E López-Jornet P. Efficacy of photobiomodulation in reducing symptomatology and improving the quality of life in patients with xerostomia and hyposalivation: a randomized controlled trial. J Clin Med. (2022) 11:3414. doi: 10.3390/jcm11123414, PMID: 35743485 PMC9225194

[58] Gür A Karakoc M Nas K Cevik R Sarac J Ataoglu S. Effects of low power laser and low dose amitriptyline therapy on clinical symptoms and quality of life in fibromyalgia: a single-blind, placebo-controlled trial. Rheumatol Int. (2002) 22:188–93. doi: 10.1007/s00296-002-0221-z, PMID: 12215864

[59] Gur A Sarac AJ Cevik R Altindag O Sarac S. Efficacy of 904 nm gallium arsenide low level laser therapy in the management of chronic myofascial pain in the neck: a double-blind and randomize-controlled trial. Lasers Surg Med. (2004) 35:229–35. doi: 10.1002/lsm.20082 15389743

[60] Iosifescu DV Norton RJ Tural U Mischoulon D Collins K McDonald E . Very low-level transcranial photobiomodulation for major depressive disorder: the Elated-3 multicenter, randomized, sham-controlled trial. J Clin Psychiatry. (2022) 83:21m14226. doi: 10.4088/JCP.21m14226, PMID: 35950904

[61] Kheradmand A Tabeie F Seif P Rezaei O Yasamy MT. Effect of low-level laser therapy (LLLT) on cognitive impairment among patients with chronic schizophrenia: a double-blind randomized placebo-controlled clinical trial. Lasers Med Sci. (2022) 37:2717–25. doi: 10.1007/s10103-022-03545-9 35314926

[62] Quah-Smith I Smith C Crawford JD Russell J. Laser acupuncture for depression: a randomised double blind controlled trial using low intensity laser intervention. J Affect Disord. (2013) 148:179–87. doi: 10.1016/j.jad.2012.11.058, PMID: 23337655

[63] Quah-Smith JI Tang WM Russell J. Laser acupuncture for mild to moderate depression in a primary care setting – a randomised controlled trial. Acupunct Med. (2005) 23:103–11. doi: 10.1136/aim.23.3.103 16259308

[64] Baldessarini RJ. The basis for amine hypotheses in affective disorders. A critical evaluation. Arch Gen Psychiatry. (1975) 32:1087–93. doi: 10.1001/archpsyc.1975.01760270019001 241308

[65] Dantzer R O'Connor JC Freund GG Johnson RW Kelley KW. From inflammation to sickness and depression: when the immune system subjugates the brain. Nat Rev Neurosci. (2008) 9:46–56. doi: 10.1038/nrn2297, PMID: 18073775 PMC2919277

[66] Maes M Yirmyia R Noraberg J Brene S Hibbeln J Perini G . The inflammatory & neurodegenerative (I&Nd) hypothesis of depression: leads for future research and new drug developments in depression. Metab Brain Dis. (2009) 24:27–53. doi: 10.1007/s11011-008-9118-1 19085093

[67] Wang B Shi H Yang B Miao Z Sun M Yang H . The mitochondrial Ahi1/GR participates the regulation on mtDNA copy numbers and brain ATP levels and modulates depressive behaviors in mice. Cell Commun Signal. (2023) 21:21. doi: 10.1186/s12964-022-01034-8, PMID: 36691038 PMC9869592

[68] Karu TI Kolyakov SF. Exact action spectra for cellular responses relevant to phototherapy. Photomed Laser Surg. (2005) 23:355–61. doi: 10.1089/pho.2005.23.355, PMID: 16144476

[69] Morries LD Cassano P Henderson TA. Treatments for traumatic brain injury with emphasis on transcranial near-infrared laser phototherapy. Neuropsychiatr Dis Treat. (2015) 11:2159–75. doi: 10.2147/ndt.S65809, PMID: 26347062 PMC4550182

[70] Salehpour F Farajdokht F Cassano P Sadigh-Eteghad S Erfani M Hamblin MR . Near-infrared photobiomodulation combined with coenzyme Q(10) for depression in a mouse model of restraint stress: reduction in oxidative stress, neuroinflammation, and apoptosis. Brain Res Bull. (2019) 144:213–22. doi: 10.1016/j.brainresbull.2018.10.010, PMID: 30385146 PMC6309497

[71] Figueiro MG Plitnick BA Lok A Jones GE Higgins P Hornick TR . Tailored lighting intervention improves measures of sleep, depression, and agitation in persons with Alzheimer's disease and related dementia living in long-term care facilities. Clin Interv Aging. (2014) 9:1527–37. doi: 10.2147/cia.S68557, PMID: 25246779 PMC4168854

[72] Ortuño-Lizarán I Esquiva G Beach TG Serrano GE Adler CH Lax P . Degeneration of human photosensitive retinal ganglion cells may explain sleep and circadian rhythms disorders in Parkinson's disease. Acta Neuropathol Commun. (2018) 6:90. doi: 10.1186/s40478-018-0596-z 30201049 PMC6130068

[73] Maiello M Losiewicz OM Bui E Spera V Hamblin MR Marques L . Transcranial photobiomodulation with near-infrared light for generalized anxiety disorder: a pilot study. Photobiomodul Photomed Laser Surg. (2019) 37:644–50. doi: 10.1089/photob.2019.4677, PMID: 31647775 PMC6818480

[74] Naeser MA Zafonte R Krengel MH Martin PI Frazier J Hamblin MR . Significant improvements in cognitive performance post-transcranial, red/near-infrared light-emitting diode treatments in chronic, mild traumatic brain injury: open-protocol study. J Neurotrauma. (2014) 31:1008–17. doi: 10.1089/neu.2013.3244 24568233 PMC4043367

[75] Saltmarche AE Naeser MA Ho KF Hamblin MR Lim L. Significant improvement in cognition in mild to moderately severe dementia cases treated with transcranial plus intranasal photobiomodulation: case series report. Photomed Laser Surg. (2017) 35:432–41. doi: 10.1089/pho.2016.4227, PMID: 28186867 PMC5568598

[76] Gonzalez-Lima F Barksdale BR Rojas JC. Mitochondrial respiration as a target for neuroprotection and cognitive enhancement. Biochem Pharmacol. (2014) 88:584–93. doi: 10.1016/j.bcp.2013.11.010, PMID: 24316434

[77] Saucedo CL Courtois EC Wade ZS Kelley MN Kheradbin N Barrett DW . Transcranial laser stimulation: mitochondrial and cerebrovascular effects in younger and older healthy adults. Brain Stimul. (2021) 14:440–9. doi: 10.1016/j.brs.2021.02.011, PMID: 33636401

[78] Pruitt T Carter C Wang X Wu A Liu H. Photobiomodulation at different wavelengths boosts mitochondrial redox metabolism and hemoglobin oxygenation: lasers vs. light-emitting diodes *in vivo*. Metabolites. (2022) 12:103. doi: 10.3390/metabo12020103, PMID: 35208178 PMC8880116

[79] Buendía D Guncay T Oyanedel M Lemus M Weinstein A Ardiles ÁO . The transcranial light therapy improves synaptic plasticity in the Alzheimer's disease mouse model. Brain Sci. (2022) 12:1272. doi: 10.3390/brainsci12101272, PMID: 36291206 PMC9599908

[80] Hamblin MR. Photobiomodulation for Alzheimer's disease: has the light dawned? Photo-Dermatology. (2019) 6:77. doi: 10.3390/photonics6030077, PMID: 31363464 PMC6664299

[81] Kolyva C Tachtsidis I Ghosh A Moroz T Cooper CE Smith M . Systematic investigation of changes in oxidized cerebral cytochrome C oxidase concentration during frontal lobe activation in healthy adults. Biomed Opt Express. (2012) 3:2550–66. doi: 10.1364/boe.3.002550 23082295 PMC3469997

[82] Jacques SL. Optical properties of biological tissues: a review. Phys Med Biol. (2013) 58:R37–61. doi: 10.1088/0031-9155/58/11/r37 23666068

[83] Tedford CE DeLapp S Jacques S Anders J. Quantitative analysis of transcranial and Intraparenchymal light penetration in human cadaver brain tissue. Lasers Surg Med. (2015) 47:312–22. doi: 10.1002/lsm.22343 25772014

[84] Kennedy SH Konarski JZ Segal ZV Lau MA Bieling PJ McIntyre RS . Differences in brain glucose metabolism between responders to Cbt and venlafaxine in a 16-week randomized controlled trial. Am J Psychiatry. (2007) 164:778–88. doi: 10.1176/ajp.2007.164.5.778 17475737

[85] Hamper M Cassano P Lombard J. Treatment of Kleine-Levin syndrome with intranasal Photobiomodulation and methylene blue. Cureus. (2021) 13:e18596. doi: 10.7759/cureus.18596, PMID: 34659921 PMC8499676

[86] Salehpour F Gholipour-Khalili S Farajdokht F Kamari F Walski T Hamblin MR . Therapeutic potential of intranasal photobiomodulation therapy for neurological and neuropsychiatric disorders: a narrative review. Rev Neurosci. (2020) 31:269–86. doi: 10.1515/revneuro-2019-0063, PMID: 31812948 PMC7138738

[87] Kamper SJ Apeldoorn AT Chiarotto A Smeets RJ Ostelo RW Guzman J . Multidisciplinary biopsychosocial rehabilitation for chronic low back pain. Cochrane Database Syst Rev. (2014) 9:Cd000963. doi: 10.1002/14651858.CD000963.pub3 PMC10945502 25180773

[88] Hennessy M Hamblin MR. Photobiomodulation and the brain: a new paradigm. J Opt. (2017) 19:013003. doi: 10.1088/2040-8986/19/1/013003, PMID: 28580093 PMC5448311

[89] Huang YY Chen AC Carroll JD Hamblin MR. Biphasic dose response in low level light therapy. Dose Response. (2009) 7:358–83. doi: 10.2203/dose-response.09-027.Hamblin 20011653 PMC2790317

[90] Huang YY Sharma SK Carroll J Hamblin MR. Biphasic dose response in low level light therapy - an update. Dose Response. (2011) 9:602–18. doi: 10.2203/dose-response.11-009.Hamblin 22461763 PMC3315174

[91] Lanzafame RJ Stadler I Kurtz AF Connelly R Peter TA Sr Brondon P . Reciprocity of exposure time and irradiance on energy density during photoradiation on wound healing in a murine pressure ulcer model. Lasers Surg Med. (2007) 39:534–42. doi: 10.1002/lsm.20519, PMID: 17659591

[92] Oron U Yaakobi T Oron A Hayam G Gepstein L Rubin O . Attenuation of infarct size in rats and dogs after myocardial infarction by low-energy laser irradiation. Lasers Surg Med. (2001) 28:204–11. doi: 10.1002/lsm.1039, PMID: 11295753

[93] Jagdeo JR Adams LE Brody NI Siegel DM. Transcranial red and near infrared light transmission in a cadaveric model. PLoS One. (2012) 7:e47460. doi: 10.1371/journal.pone.0047460, PMID: 23077622 PMC3471828

[94] Hwang MH Son HG Lee JW Yoo CM Shin JH Nam HG . Photobiomodulation of extracellular matrix enzymes in human nucleus pulposus cells as a potential treatment for intervertebral disk degeneration. Sci Rep. (2018) 8:11654. doi: 10.1038/s41598-018-30185-3, PMID: 30076336 PMC6076240

