# Correction: Photobiomodulation improves depression symptoms: a systematic review and meta-analysis of randomized controlled trials

**DOI:** 10.3389/fpsyt.2025.1671091

**Published:** 2025-09-10

**Authors:** Qipei Ji, Shichang Yan, Jilin Ding, Xin Zeng, Zhixiang Liu, Tianqi Zhou, Zhuorao Wu, Wei Wei, Huaqiang Li, Shuangyue Liu, Shuangchun Ai

**Affiliations:** ^1^ School of Health Preservation and Rehabilitation, Chengdu University of Traditional Chinese Medicine, Chengdu, China; ^2^ Department of Rehabilitation, Mianyang Hospital of Traditional Chinese Medicine, Mianyang, China; ^3^ Department of Endocrinology and Metabolism, The Affiliated Hospital of Southwest Medical University, Luzhou, China

**Keywords:** photobiomodulation, t-PBM, s-PBM, low-level laser therapy, depression, sleep, meta-analysis

In the **Abstract**, there was an error in the *Results*. It was previously reported “The best improvement for t-PBM was achieved using a wavelength of 823 nm, fluence of 10–100 J/cm^2^, irradiance of 50–100 mW/cm^2^, irradiance time of 30 min, treatment frequency < 3/week, and number of treatments > 15 times.” This has been corrected to: “The best improvement for t-PBM was achieved using a wavelength of 823 nm, fluence of 10–100 J/cm^2^, irradiance ≤ 50 mW/cm^2^, irradiance time of 30 min, treatment frequency < 3/week, and number of treatments > 15 times.”

There was an error in **5. Conclusion**, Paragraph 1. “The irradiance selection of t-PBM was displayed as “50–100 mW/cm^2^”. The correct sentence is: “t-PBM wavelength selection 823 nm, fluence selection 10–100 J/cm^2^, irradiance selection ≤ 50  mW/cm^2^, irradiance time selection 30 min, treatment frequency < 3/week, number of treatments > 15 times”

The original version of this article has been updated.

