# Supplementary material for: Photobiomodulation improves depression symptoms: a systematic review and meta-analysis of randomized controlled trials
Source: Front Psychiatry. 2024 Jan 31;14:1267415. doi: 10.3389/fpsyt.2023.1267415 (PMC10866010; doi:10.3389/fpsyt.2023.1267415)
Supplement: Supplementary file 2 [file Data_Sheet_2.docx]

**Search strategies of electronic databases**

| **Database** | **Search strategy** |
| --- | --- |
| Pubmed | #1 Depression [MeSH]  #2 Depression*[tiab] OR Depressive Symptoms*[tiab] OR Depressive Symptom *[tiab] OR Symptom, Depressive*[tiab] OR Symptoms, Depressive*[tiab] OR Emotional Depression*[tiab] OR Depression, Emotional*[tiab]  #3 #1 OR #2  #4 Low-Level Light Therapy [MeSH]  #5 Low-Power Laser Therapies*[tiab] OR Low-Level Laser Therapy*[tiab] OR Low Level Laser Therapy*[tiab] OR Low Power Laser Irradiation*[tiab] OR Low-Power Laser Irradiation*[tiab] OR Laser Biostimulation*[tiab] OR Biostimulation, Laser*[tiab] OR Laser Phototherapy*[tiab] OR Phototherapy, Laser*[tiab] OR low-level light therapy*[tiab] OR laser therapy*[tiab] OR phototherapy*[tiab] OR transcranial laser*[tiab] OR photobiomodulation*[tiab] OR Transcranial Photobiomodulation *[tiab] OR PBM*[tiab] OR Transcranial Low-level Laser Therapy*[tiab]  #6 #4 OR #5  #7 Randomized controlled trial*[tiab] OR randomized*[tiab] OR placebo*[tiab]  #8 #3 AND #6 AND #7 |
| Embase | #1 'depression'/exp OR depression  #2 depression:ab,ti OR 'depressive symptoms':ab,ti OR 'depressive symptom':ab,ti OR 'symptom, depressive':ab,ti OR 'symptoms, depressive':ab,ti OR 'emotional depression':ab,ti OR 'depression, emotional':ab,ti  #3 #1 OR #2  #4 'low level' AND ('light'/exp OR light) AND ('therapy'/exp OR therapy)  #5 'low-power laser therapies':ab,ti OR 'low-level laser therapy':ab,ti OR 'low level laser therapy':ab,ti OR 'low power laser irradiation':ab,ti OR 'low-power laser irradiation':ab,ti OR 'laser biostimulation':ab,ti OR 'biostimulation, laser':ab,ti OR 'laser phototherapy':ab,ti OR 'phototherapy, laser':ab,ti OR 'low-level light therapy':ab,ti OR 'laser therapy':ab,ti OR phototherapy:ab,ti OR 'transcranial laser':ab,ti OR photobiomodulation:ab,ti OR 'transcranial photobiomodulation':ab,ti OR pbm:ab,ti OR 'transcranial low-level laser therapy':ab,ti  #6 #4 OR #5  #7 'randomized controlled trial randomized':ab,ti OR placebo:ab,ti OR rct:ab,ti  #8 #3 AND #6 AND #7 |
| Cochrane Library | #1 MeSH descriptor: [Depression] explode all trees  #2 (Depression OR Depressive Symptoms OR Depressive Symptom OR Symptom, Depressive OR Symptoms, Depressive OR Emotional Depression OR Depression, Emotional):ti,ab,kw (Word variations have been searched)  #3 MeSH descriptor: [Low-Level Light Therapy] explode all trees  #4 (Low-Power Laser Therapies OR Low-Level Laser Therapy OR Low Level Laser Therapy OR Low Power Laser Irradiation OR Low-Power Laser Irradiation OR Laser Biostimulation OR Biostimulation, Laser OR Laser Phototherapy OR Phototherapy, Laser OR low-level light therapy OR laser therapy OR phototherapy OR transcranial laser OR photobiomodulation OR Transcranial Photobiomodulation OR PBM OR Transcranial Low-level Laser Therapy):ti,ab,kw (Word variations have been searched)  #5 #1 OR #2  #6 #3 OR #4  #7 #5 AND #6 |
| Web of science | #1 TS = (Depression OR Depressive Symptoms OR Depressive Symptom OR Symptom, Depressive OR Symptoms, Depressive OR Emotional Depression OR Depression, Emotional)  #2 TS = (Low-Power Laser Therapies OR Low-Level Laser Therapy OR Low Level Laser Therapy OR Low Power Laser Irradiation OR Low-Power Laser Irradiation OR Laser Biostimulation OR Biostimulation, Laser OR Laser Phototherapy OR Phototherapy, Laser OR low-level light therapy OR laser therapy OR phototherapy OR transcranial laser OR photobiomodulation OR Transcranial Photobiomodulation OR PBM OR Transcranial Low-level Laser Therapy)  #3 #1 AND #2  #4 TS = (Randomized controlled trial Randomized OR Placebo OR RCT)  #5 #3 AND #4 |
| Psyclnfo | #1 SU Depression OR Depressive Symptoms OR Depressive Symptom OR Symptom,Depressive OR Symptoms, Depressive OR Emotional Depression OR Depression, Emotional  #2 SU Low-Power Laser Therapies OR Low-Level Laser Therapy OR Low Level Laser Therapy OR Low Power Laser Irradiation OR Low-Power Laser Irradiation OR Laser Biostimulation OR Biostimulation, Laser OR Laser Phototherapy OR Phototherapy, Laser OR low-level lighttherapy OR laser therapy OR phototherapy OR transcranial laser OR photobiomodulation OR Transcranial Photobiomodulation OR PBM OR Transcranial Low-level Laser Therapy OR Near-infrared OR near-infrared imaging system OR near-infrared light OR NIR OR near-infrared laser  #3 SU Randomized controlled trial OR Randomized OR Placebo OR RCT  #4 #1 AND #2 AND #3 |
| ScienceDirect | #1"Depression"  #2 "Low-Power Laser Therapies" OR " Photobiomodulation" OR "Low-Level Laser Therapy" OR "Near-infrared" OR "NIR"  #3 "Randomized controlled trial" OR "RCT"  #4 #1 AND #2 AND #3 |
| CNKI | #1（主题：抑郁 + 抑郁症 + 抑郁障碍 + 抑郁症状 + 抑郁情绪(精确)）  #2（主题：光生物调节 + 低水平激光疗法 + 低水平激光疗法 + PBM + 近红外光 + 低水平光治疗(精确)）  #3 #1 AND #2 |
| VIP | #1 题名或关键词=抑郁+抑郁症+抑郁障碍+抑郁症状+抑郁情绪  #2题名或关键词= 光生物调节+低水平激光疗法+低水平激光疗法+PBM+近红外光+低水平光治疗  #3 #1 AND #2 |
| Sinomed | #1 ( "抑郁"[常用字段:智能] OR "抑郁症"[常用字段:智能] OR "抑郁障碍"[常用字段:智能] OR "抑郁症状"[常用字段:智能] OR "抑郁情绪"[常用字段:智能])  #2 ( "光生物调节"[常用字段:智能] OR "低水平激光疗法"[常用字段:智能] OR "低水平激光疗法"[常用字段:智能] OR " PBM "[常用字段:智能] OR“近红外光"[常用字段:智能])  #3 #1 AND #2 |
| Wangfang | #1 (主题=抑郁) AND 题名或关键词=(抑郁 or 抑郁症 or 抑郁障碍 or 抑郁症状 or 抑郁情绪)  #2 (((题名或关键词=(光生物调节 or 低水平激光疗法 or 低水平激光疗法)) OR 题名或关键词=(PBM or 近红外光 or 低水平光治疗))) AND ((题名或关键词=(光生物调节 or 低水平激光疗法 or 低水平激光疗法)) OR 题名或关键词=(PBM or 近红外光 or 低水平光治疗))  #3 #1 AND #2 |
