# Supplementary material for: Photobiomodulation improves depression symptoms: a systematic review and meta-analysis of randomized controlled trials
Source: Front Psychiatry. 2024 Jan 31;14:1267415. doi: 10.3389/fpsyt.2023.1267415 (PMC10866010; doi:10.3389/fpsyt.2023.1267415)
Supplement: Supplementary file 3 [file Data_Sheet_3.docx]

**Author(s): Qipei Ji**
**Date:** 2023-10-22
**Question:** Should PBM be used for depression?
**Settings:**
**Bibliography:** Cochrane Database of Systematic Reviews [Year], Issue [Issue].

| **Quality assessment** | | | | | | | **No of patients** | | **Effect** | | **Quality** | **Importance** |
| --- | --- | --- | --- | --- | --- | --- | --- | --- | --- | --- | --- | --- |
|  |  |  |  |  |  |  |  |  |  |  |  |  |
| **No of studies** | **Design** | **Risk of bias** | **Inconsistency** | **Indirectness** | **Imprecision** | **Other considerations** | **TPBM** | **Control** | **Relative (95% CI)** | **Absolute** |  |  |
| **Depression (Better indicated by lower values)** | | | | | | | | | | | | |
| 11 | randomised trials | no serious risk of bias | no serious inconsistency | no serious indirectness | serious^1^ | none | 200 | 207 | - | SMD 0.55 lower (0.75 to 0.35 lower) | ⊕⊕⊕O MODERATE | CRITICAL |
| **sleep (Better indicated by lower values)** | | | | | | | | | | | | |
| 2 | randomised trials | no serious risk of bias | very serious^1,2^ | no serious indirectness | serious | none | 37 | 38 | - | MD 0.82 lower (2.41 lower to 0.77 higher) | ⊕OOO VERY LOW | NOT IMPORTANT |

^1^ The sample size of the included study was too small
^2^ The confidence interval is too wide
