# Supplementary material for: Photobiomodulation improves depression symptoms: a systematic review and meta-analysis of randomized controlled trials
Source: Front Psychiatry. 2024 Jan 31;14:1267415. doi: 10.3389/fpsyt.2023.1267415 (PMC10866010; doi:10.3389/fpsyt.2023.1267415)
Supplement: Supplementary file 4 [file Data_Sheet_4.docx]

**Subgroup analyses of depression outcome (t-PBM and s-PBM)**

**t-PBM vs s-PBM**

**
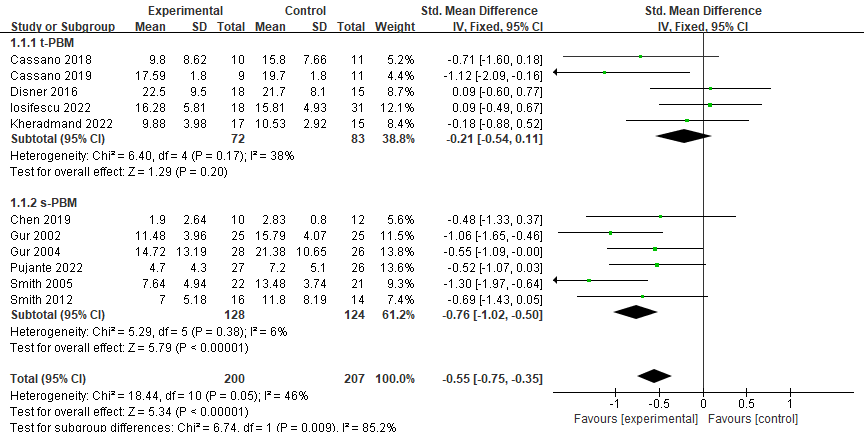
**

**t-PBM**

**age**

**
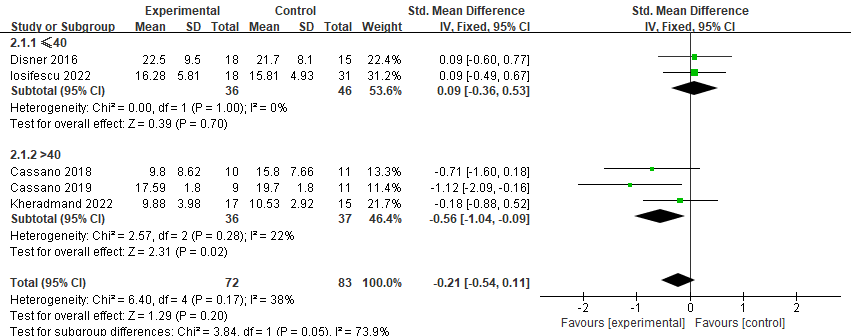
**

**Light source**

**
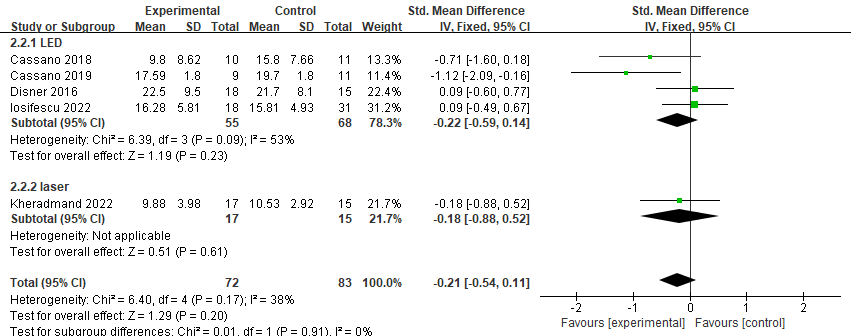
**

**irradiance time**

**
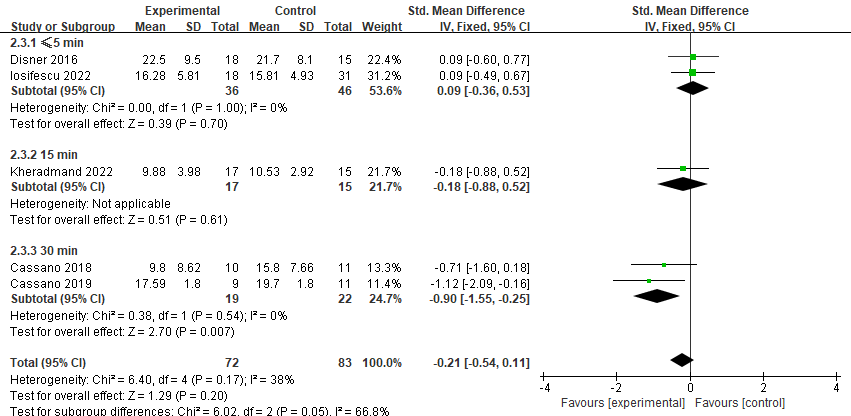
**

**Wavelength**

**
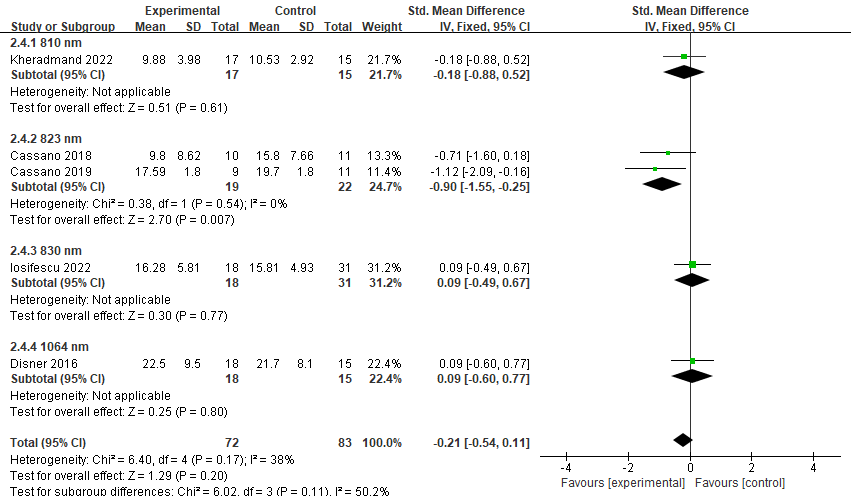
**

**fluence**

**
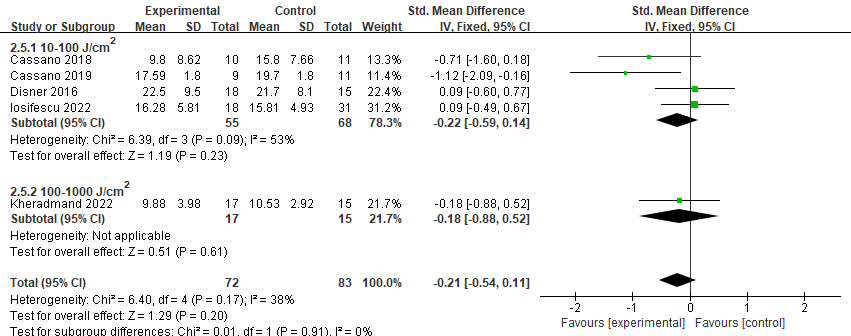
**

**irradiance**

**
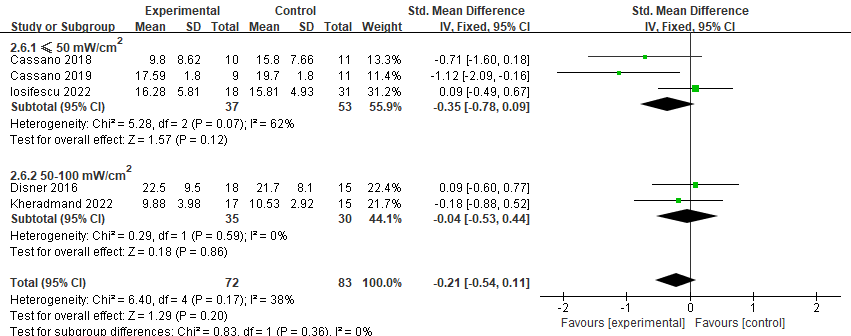
**

**treatment frequency**

**
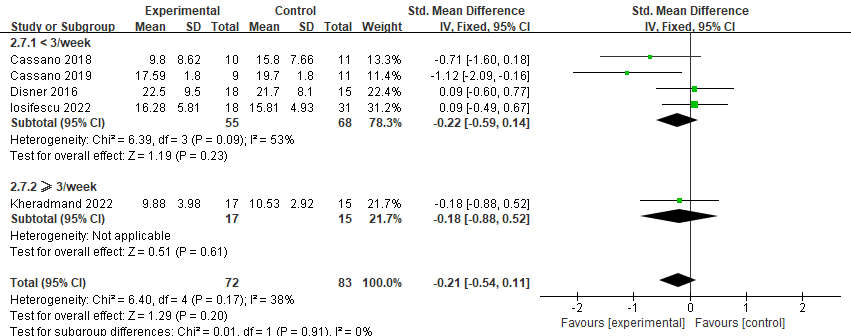
**

**number of treatments**

**
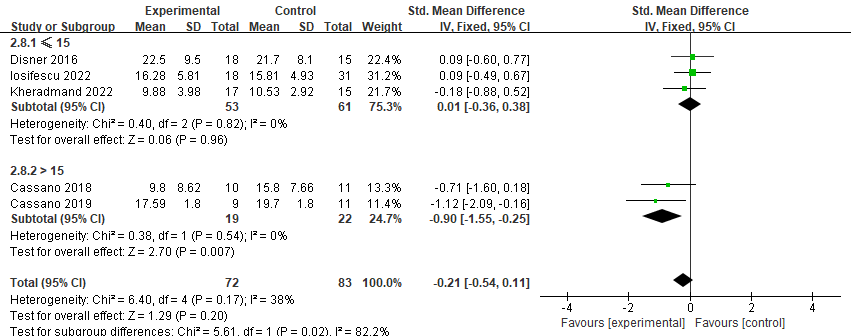
**

**s-PBM**

**age**

**
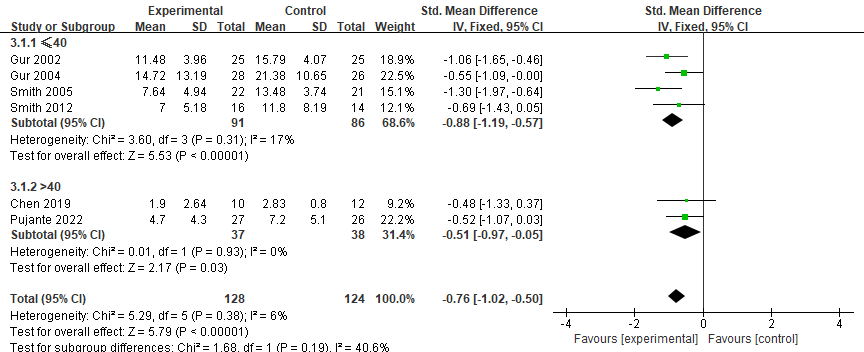
**

**irradiation site**

**
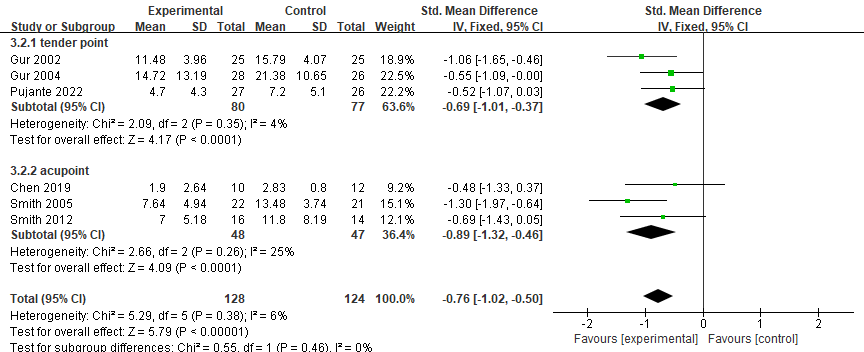
**

**Light source**

**
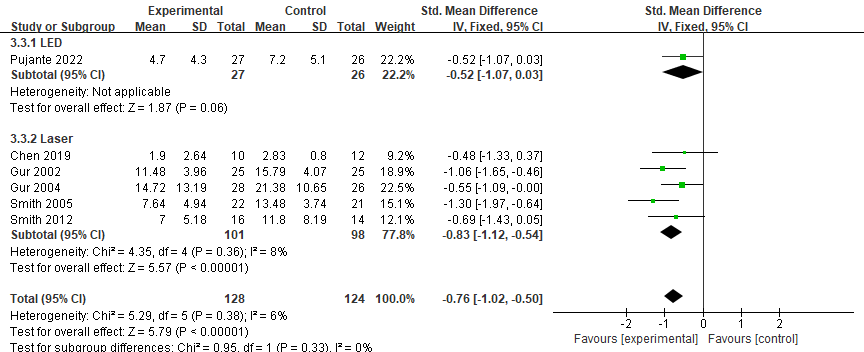
**

**irradiance time**

**
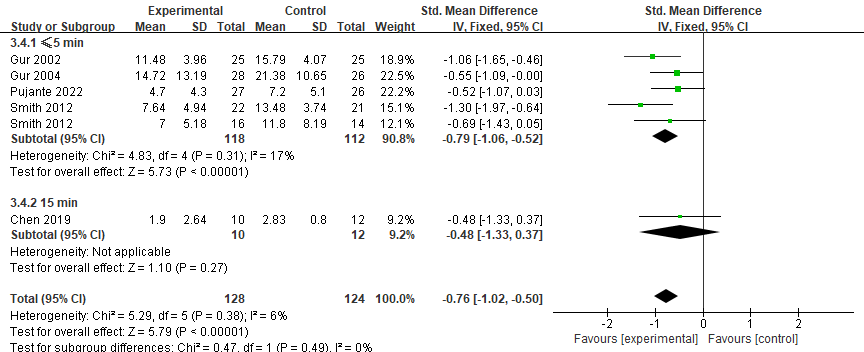
**

**Wavelength**

**
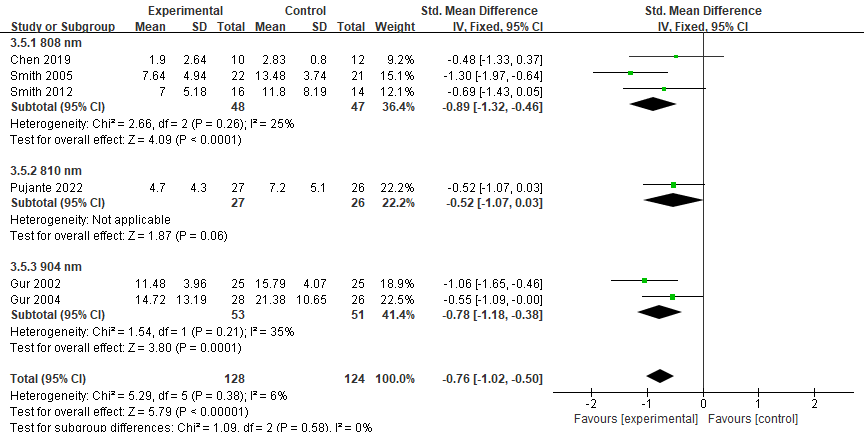
**

**Fluence**

**
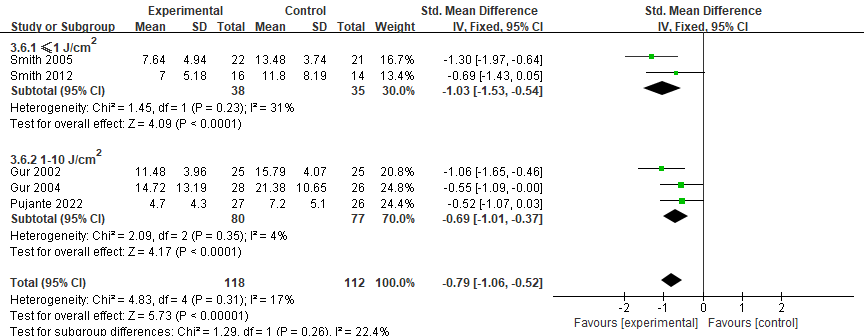
**

**Irradiance**

**
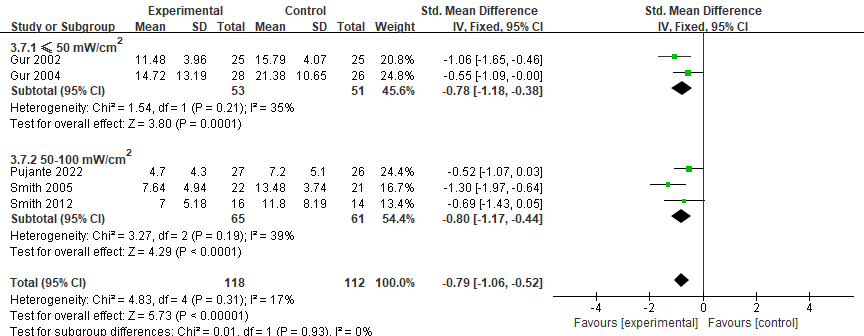
**

**treatment frequency**

**
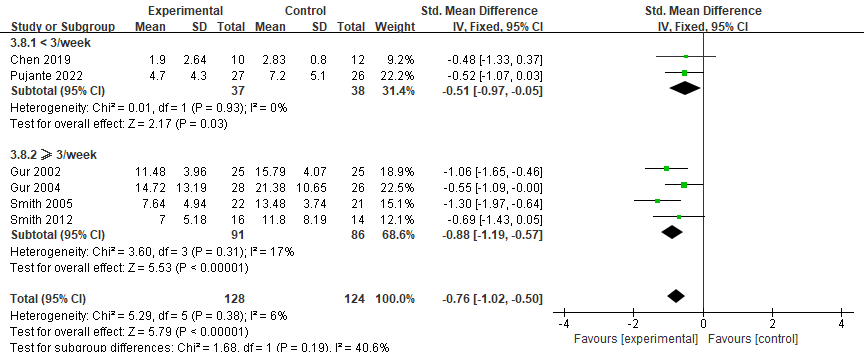
**

**number of treatments**

**
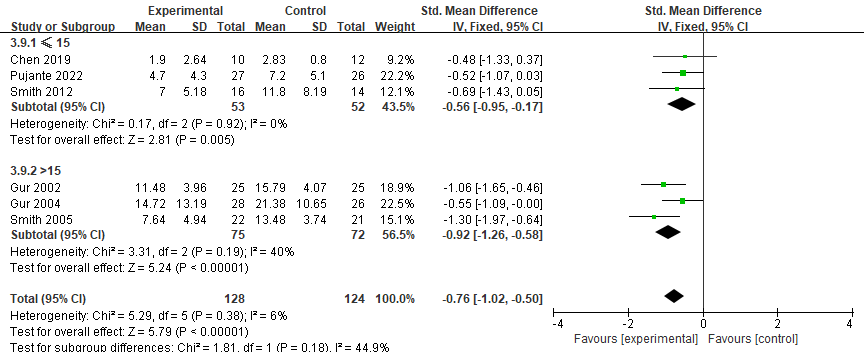
**
